# Role of rich phenolics and betanin profiles from *Opuntia ficus-indica* fruits in the prevention of diabetic complications using metabolomics study

**DOI:** 10.1038/s41598-024-81874-1

**Published:** 2025-02-17

**Authors:** Mona A. Mohammed, Souad E. El-Gengaihi, Yousreya A. Maklad, Marwa E. Shabana, Hanan Naeim Attia

**Affiliations:** 1 Medicinal and Aromatic Plants Research Department, Pharmaceutical Industries Research Institute, National Research Centre, Giza, Egypt; 2 Medicinal and Pharmaceutical Chemistry Department (Pharmacology Group), Pharmaceutical Industries Research Institute, National Research Centre, Giza, Egypt; 3 Pathology Department, Medical Research and Clinical Studies Institute, National Research Centre, Giza, Egypt

**Keywords:** UPLC/HESI-MS/MS, *Opuntia* spp, Phenolics, Betanin, Antidiabetic, Oxidative stress, Streptozotocin, Adiponectin, Cholesterol, Biochemistry, Biotechnology, Plant sciences

## Abstract

*Opuntia ficus-indica* red fruit (OFI-RF) is a member of the *Cactaceae* family and native to South America. Phytochemical evaluation of the plant has revealed variable bioactive components; therefore, this study explored the medicinal value of butanol (BE) and ethylacetate extracts (EE) by evaluating their antidiabetic, antioxidant and antihypercholesterolemic properties. Selected solvents were used for phytochemical extraction according to established protocols, and then pharmacological effects of phenolic and betanin-rich extracts were evaluated. Results indicated that butanol was the most effective solvent for extracting polyphenolics followed by ethyl acetate, yielding: 148.91 ± 0.95 and 110.96 ± 0.61 μg/g, respectively. Identification analysis of OFI-RF using UPLC/HESI-MS/MS revealed a diverse range of 101 metabolites, including polyphenolics (phenolic acids, phenolic glycosides, flavanols, flavanonols, flavonoids and biflavonoids), alkaloids, pyridine, betalains, coumarins, vitamins, fatty acids and other therapeutic compounds. Biological studies (in vitro and in vivo) demonstrated that both EE and BE exhibited significant antidiabetic, antioxidant and antihypercholestremic activities. These findings were further supported via histopathological examination.

## Introduction

*Opuntia ficus-indica* red fruit OFI-RF is a member of the *Cactaceae* family that is widely distributed globally. The OFI, originally from South America (specifically Mexico), was introduced to Spain by Christopher Columbus in 1493 and later naturalized across the Mediterranean region and northern Africa. Due to its genetic diversity, OFI is highly adaptable and widely distributed, thriving in varied climates across north, central, and south America, northern and southern Africa, the middle east, Australia, India, and Mediterranean countries^1,2^. Mexico and Italy are the primary producers and consumers of OFI. Mexico holds the highest genetic diversity of the species and, along with Italy, accounts for 70% and 3.3% of the 590,000 ha cultivated globally, respectively^3,4^. In Mexico, cladodes are the fifth most consumed vegetable, and prickly pear ranks as the third most consumed fruit^5,6^. This difference in appearance is attributed to unequal distribution of the pigment color and region of growth^6^. A high concentration of the polyphenolics^7^, stilbenes^8^, flavanols^9^, flavanonols^9^, flavonoids^7^, lignans^10^, suberin-acids^10^, as well as betalains^7^, alkaloids^11^, and pyridine carboxylic acids^10,12^ has been reported. These substances improve blood flow, reduce the risk of heart disease, enhance cognitive performance^13^, minimize neurological diseases, and reduce cell death induced by oxidative stress and free radicals^14,15^.

Type 2 diabetes mellitus (T2DM) affects over 537 million people worldwide and is one of the chronic diseases with the highest growth rate along with the obesity pandemic^16^. This debilitating disease significantly raises costs of healthcare system; hence it is necessary to explore innovative treatments and preventive measures that could retard its deteriorating effects globally. Nevertheless, therapeutic methods are generally affected by cost, accessibility, compliance, and adverse effects that may pose hazard, particularly in elderly population^17^.

It has long been known that effective nutritional therapies can improve glycemic control, such as the Mediterranean diet^18^ and usage of functional food. Functional food has been reported to assist in the management of a number of health problems, including hyperglycemia. The use of OFI-RF and related products lowered post-prandial glucose levels in numerous trials possibly by altering glucose absorption thus, reducing its release into the blood circulation. High fiber content of OFI-RF may also have a key role in glycemic control^19^. Mucus that humans generally cannot digest is a type of dietary fiber found in this genus as well. Soft stems, or nopalitos, are commonly consumed in Mexico to treat diabetes. Frati-Munari et al. studied the hypoglycemic effect of cactus pads, which inhibited glucose absorption in the intestine and led to a significant reduction in blood glucose levels within 120–180 min after ingestion of *Opuntia ficus-indica*^20^. Trejo-González et al. suggested that the blood glucose-lowering effects of a purified *Opuntia* extract in diabetic mice may be due to high dietary fiber content^21^. The aim of this comprehensive study was designed for phytochemical characterization and pharmacological evaluation of OFI-RF selected extracts against diabetes and its related complications. Additionally, histopathological examination of pancreatic sections was conducted for further assessment of findings.

## Materials and methods

### Phytochemical studies

#### Chemicals

Trolox, DPPH, ABTS, and Folin-Ciocalteu reagent were purchased from Sigma Aldrich. All other chemicals and solvents displayed a high level of purity and conformed to the standards for analytical grade.

#### Plant materials and extraction

*Opuntia ficus-indica *fruit was collected from a private Sekem farm in El Salam City, Cairo, Egypt. A specimen was deposited at the herbarium unit of the National Research Centre under Number M248. Three kilograms of fresh fruit were cut into slices, dried by lyophilization, and milled to a particle size of 75–180 μm, then exhaustively extracted by maceration with 5 L of methanol at room temperature (25–30 °C) in five repeated cycles. The substance underwent filtration and concentration at 45 °C utilizing a rotary evaporator under decreased pressure. Subsequently, the crude methanol extract residue was suspended in 1 L water, allowed to stand overnight, and consecutively separated using 0.7 L petroleum ether (PE), 0.7 L chloroform (CE), 0.7 L ethylacetate (EE), and finally 0.7 L butanol (BE) five times repeatedly for each solvent in ascending order of polarity^22^. Pooled QC samples (mixed EE, TE and BE extract) are used to condition the analytical platform, assess intra-study reproducibility, and correct for systematic errors through mathematical adjustments of bioinformatics processing^23^.

#### Determination of total flavonoids, phenols in different *Opuntia spp.* extracts

Flavonoids and phenols were quantified in various extracts using the established methodologies^24^. The mean ± standard error of the mean (s.e.m) using t-test analysis by GraphPad Prism software (USA, version 8.0.1). The level of statistical significance was adjusted to *p* value of less than 0.05.

#### Metabolomics and molecular networks of secondary metabolites from *Opuntia spp* fruits by using LC/MS/MS via MS-Dial

The UPLC system (Acquity, Waters, Milford, USA) was connected to a Q Exactive hybrid MS/MS quadrupole-Orbitrap mass spectrometer. The chromatographic separation protocol employed in this setup utilized an aqueous solution acidified with 0.1% formic acid (referred to as solvent A) along with acetonitrile (referred to as solvent B), with a mobile phase flow rate set at 0.4 ml/min^25^. Specifically, the gradient used was as follows: from 0 to 7 min, there was a transition from 50% A to 50% B; from 7 to 10 min, the composition changed to 98% B and these conditions were sustained for 15 min. Subsequently, from 10 to 13 min, the system was adjusted to 95% A, before returning to the initial conditions by 15 min. Following this, a re-equilibration period of 2 min was implemented using the BEH shield C18 column^26,27^. The selected potential metabolites were searched and identified by comparing their fragmentation patterns and retention indices (RI) by those available in Ms-Dial, Golm, KNApSAcK, Fiehn BinBase and RIKEN databases^28^.

Several factors affect HPLC analysis of phenolics, including sample preparation, mobile phase, column type, and detector choice. Utilizing MS/MS data from literature, along with a hybrid database and search algorithm, we developed an online MS/MS database to improve metabolite identification. MS-DIAL and MetaboAnalyst were used for MS/MS fragmentation analysis and to identify the complete profile of the OFI-RF plant.

### Pharmacological studies

Biological evaluation was conducted using selected extracts with the highest polyphenolics and flavonoids content according to results of the phytochemical characterization.

#### In vitro study

##### Antidiabetic activity

*(i) α-Amylase inhibitory activity*: Inhibitory activity against α-amylase enzyme was determined spectrophotometrically at 595 nm according to method described by Xiao et al.^29^ against blank. Acarbose was used as standard reference.

*(ii) α-Glucosidase inhibitory activity*: Inhibition of α-glucosidase enzyme activity was measured spectrophotometrically 405 nm according to the method described by Elya et al.^30^ against blank and acarbose (standard reference).

##### DPPH and ABTS antioxidant activity

The DPPH^•^ and ABTS^•+^ radical scavenging activity assays were utilized to evaluate the antioxidant potential of *Opuntia* spp extracts^22^ and their fractions including; water (H_2_O), butanol (BE), ethylacetate (EE), chloroform (CE), petroleum ether (PE), and total extract (TE), at various concentrations ranging from 30, 20, 10, 5, 2.5, 1.5, 1 and 0.5 μg/mL. Standard references (ascorbic acid and trolox) were employed during the analysis, which was conducted in triplicates as depicted in Fig. 3. The assessment of antioxidant activity was based on the radical scavenging model using 1,1-diphenyl-2-picrylhydrazyl (DPPH, 250 mM) according to the methodology explained by Shimada et al.^31^.

In the second method, ABTS^•+^ was dissolved in water to achieve a concentration of 7 mM. The generation of ABTS^•+^ radical cation (ABTS^•+^) was accomplished by mixing the ABTS^•+^ stock solution with 2.45 mM potassium persulfate. Each assay included appropriate solvent blanks, consistent with the protocol by Dinkova-Kostova et al.^32^. The percentage of inhibition for both DPPH^•^ and ABTS^•+^ radicals was determined using the formula: % Inhibition = [(A control − A sample)/A control] × 100, where A represents the absorbance at 517 nm for DPPH^•^ and 734 nm for ABTS^•+^^33^.

#### In vivo study

##### Animals

Adult male Wistar rats (200–250 g) were purchased from the animal facility of the National Research Centre. They were acclimatized one week before the investigation period and kept under conventional laboratory conditions with 12 h light/dark exposure^34^. Standard pellet diet and free access to water was provided throughout the study (3 weeks). All animal procedures follow regulations approved by the Ethics Committee (National Research Centre/ no. 20140)^35^ and in accordance with the recommendations for the proper care and use of laboratory animals (Percie et al.^36^, The ARRIVE guidelines, 2020**).** ARRIVE guidelines (https://arriveguidelines.org).

##### Drugs and chemicals

Streptozotocin (STZ; Sigma, USA) and Gliclazide (GZ; Amoun, Egypt) were used in the study. Diagnostic enzyme kits were purchased from MD (Italy), MyBiosource (San Diego, USA), PicoKine ELISA Kit (Pleasanton, USA) for the determination of glucose, insulin and adiponectin in rat serum, respectively. Estimation of oxidative stress and lipid peroxidation was performed using SOD kits from MyBiosource (San Diego, USA) and rat MDA ELISA kit (LifeSpan Bioscience Inc., USA). Lipid profile was assessed using cholesterol and HDL assay kits (Biochain, California, USA).

##### Experimental design

Animals were randomly assigned to six groups of eight animals each. They received their daily oral treatments for three successive weeks as follows:

Group1: Normal animals received distilled water and citrate buffer (0.1 M, pH 4.5) orally and intraperitoneally, respectively, and served as negative control. Groups (2–6) each received a single intraperitoneal dose of 45 mg/kg STZ^37^ dissolved in cold citrate buffer (0.1 M, pH 4.5). After confirmation of diabetes using a glucose meter device (Bionime, Switzerland), group 2 served as the positive control untreated group. Group 3 diabetic rats received oral reference drug; GZ (10 mg/kg)^38^. Groups 4–6 diabetic animals were given 200 mg/kg of OP extracts; total extract (TE), butanol extract (BE) and ethylacetate extract (EE), respectively. All animals were euthanized using isoflurane inhalation in anesthesia chamber. Blood was collected from orbital venous plexus, centrifuged for serum separation, then stored in − 80 °C for biochemical analyses after termination of the study. After decapitation of rats, pancreas was separated and dissected for histological examination.

##### Determination of carbohydrate metabolic changes

Metabolic changes are closely associated with STZ-induced diabetes; hence %body weight change, serum glucose, insulin and adiponectin were determined according to the manufacturer’s instructions provided within the kits.

##### Estimation of oxidative stress

SOD in rat serum was determined spectrophotometrically to assess oxidative stress in vivo as per the manufacturer’s instructions. Optical density reading was proportional to the enzyme activity in sample/ standard and was expressed as U/mL.

##### Lipid peroxidation

Lipid peroxidation was estimated as MDA concentration in rat serum and expressed as ng/mL according to the manufacturer’s manual provided.

##### Lipid profile

Cholesterol and HDL were measured using procedural steps in the manual provided within Biochain’s Assay kit (USA). Serum LDL value was determined by a method described by de Cordova et al.^39^.

### Histopathological study

#### Histopathology examination

Pancreatic tissue was dissected from rats, washed with a solution of normal saline, and subsequently separated from adipose tissue. It was then immersed in buffered 10% formalin for fixation. The process of dehydrating the samples involved a series of incremental dilutions of ethyl alcohol. Following dehydration, the samples underwent a clearing process in xylene before being embedded in paraffin at a temperature of 56 °C within an incubator for duration of 24 h. Subsequently, paraffin blocks were meticulously prepared and sliced into sections with a thickness of 4 μm. These sections were mounted onto glass slides, subjected to deparaffinization, and then stained with hematoxylin and eosin for histological analysis. Additionally, 4 μm sections were treated with Masson’s trichrome stain to evaluate fibrotic changes. The resulting images were scrutinized and captured using a digital camera (Microscope Digital Camera DP 70, Tokyo) and processed utilizing Adobe Photoshop, Version 8.0.

#### Histomorphometric quantitative analysis

Extent of fibrosis and its distribution was assessed through histomorphometric quantitative analysis utilizing an image analysis system at the pathology laboratory of the Medical Research Centre of Excellence (MRCE) unit, National Research Centre. The evaluation employed the image analysis system Leica Qwin DW3000 by LEICA Imaging Systems Ltd, Cambridge, England, comprising a Leica DM-LB microscope with JVC color video camera that is connected to a computer system. The system was meticulously configured for fibrosis assessment, with the extent of fibrosis quantified as a percentage of the total examined pancreatic area. This percentage was calculated as an area per field in micrometer square, area fraction and area percentage using the interactive measurement software of the system. The findings were automatically displayed on the monitor in tabular format, featuring total, mean, standard deviation, standard error, minimum area, and maximum area measured.

### Statistical analysis

The results of biological investigations were reported as the mean ± standard error of the mean (s.e.m) using one-way analysis of variance (one-way ANOVA), followed by Tukey’s post hoc analysis using GraphPad Prism software (USA, version 5). The level of statistical significance was adjusted to *p*-value of less than 0.05.

## Results and discussion

### Phytochemical study

#### Estimation of total flavonoids and phenolics

**Fig. 1:**
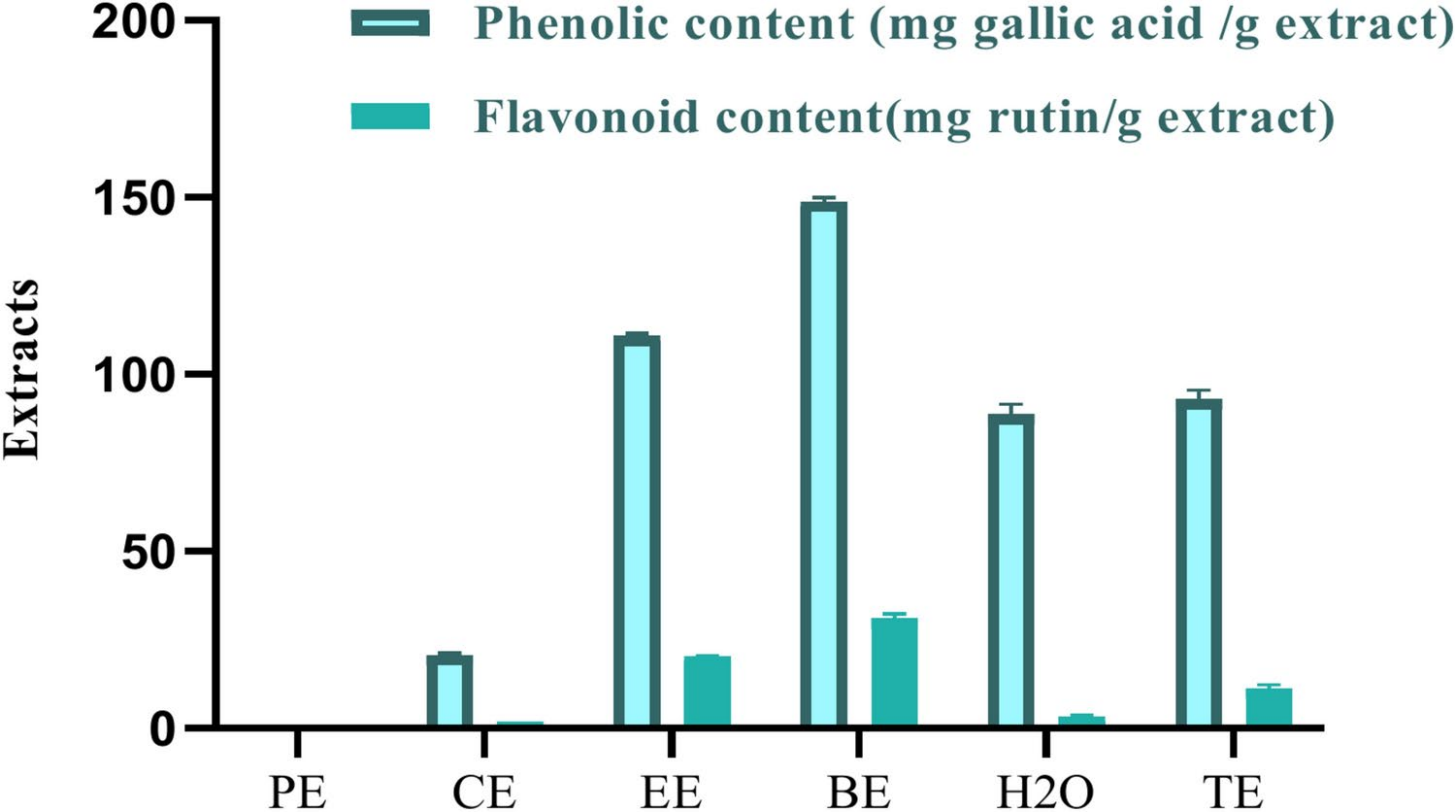
Total phenolic and flavonoid content.

The total flavonoids and polyphenolics are widely recognized for their antioxidative^40^ and antidiabetic properties. They were quantified in five fractions and a comprehensive extract was derived from *Opuntia* spp. fruits, as illustrated in Fig. 1 and Table S1. The quantification of total phenolic and flavonoid content, along with the evaluation of anti-diabetic enzyme inhibition, revealed significant variability across the five fractions of *Opuntia* spp. extracts. Among the fractions, the butanol (BE) extract exhibited the highest levels of phenolic content (148.91 ± 0.95 mg gallic acid/g extract) and flavonoid content (31.10 ± 1.07 mg rutin/g extract), indicating its potential as a rich source of these bioactive compounds in Fig. 1. This extract also demonstrated strong inhibitory activity against both α-amylase (IC50: 6.51 ± 0.24 µg/mL) and α-glucosidase (IC_50_: 5.58 ± 0.45 µg/mL), suggesting its potential for managing blood glucose levels in Fig. 5. The ethyl acetate (EE) extract, while lower in phenolic (110.96 ± 0.61 mg gallic acid/g extract) and flavonoid content (20.41 ± 0.16 mg rutin/g extract), still exhibited promising inhibitory effects on both enzymes (α-amylase IC50: 7.51 ± 0.17 µg/mL and α-glucosidase IC_50_: 8.33 ± 0.02 µg/mL). Conversely, the petroleum ether (PE) extract contained no detectable phenolics or flavonoids, and displayed the weakest inhibitory activity against both enzymes (α-amylase IC50: 50.28 ± 0.03 µg/mL and α-glucosidase IC_50_: 55.33 ± 1.00 µg/mL), supporting the conclusion that the presence of phenolic and flavonoid compounds is closely linked to the anti-diabetic activity.

The chloroform (CE) and water (H_2_O) extracts demonstrated moderate phenolic and flavonoid content and enzyme inhibition activities, with the chloroform extract (phenolic: 20.67 ± 0.56 mg gallic acid/g extract, flavonoid: 1.89 ± 0.05 mg rutin/g extract) showing better inhibition of α-amylase (IC_50_: 45.67 ± 0.57 µg/mL) compared to α-glucosidase (IC_50_: 50.73 ± 0.55 µg/mL). The water extract (phenolic: 88.67 ± 2.87 mg gallic acid/g extract, flavonoid: 3.38 ± 0.23 mg rutin/g extract) exhibited more modest enzyme inhibition (α-amylase IC_50_: 31.06 ± 1.33 µg/mL and α-glucosidase IC_50_: 29.76 ± 0.51 µg/mL). The total alcohol (TE) extract showed intermediate phenolic (93.07 ± 2.57 mg gallic acid/g extract) and flavonoid (11.47 ± 0.74 mg rutin/g extract) content, along with moderate enzyme inhibition (α-amylase IC50: 10.29 ± 0.33 µg/mL and α-glucosidase IC50: 10.99 ± 0.58 µg/mL).

These findings indicate that *Opuntia* spp. fruits, particularly the butanol and ethyl acetate extracts, have significant anti-diabetic potential, which could be attributed to their rich phenolic and flavonoid content. This supports their traditional use in managing diabetes and highlights the importance of these bioactive compounds in combating diabetes-related complications.

#### Metabolomic profiles molecular networks of successive extraction of from OFI-RF fruits by using LC/MS/MS and MS-Dial

**Fig. 2:**
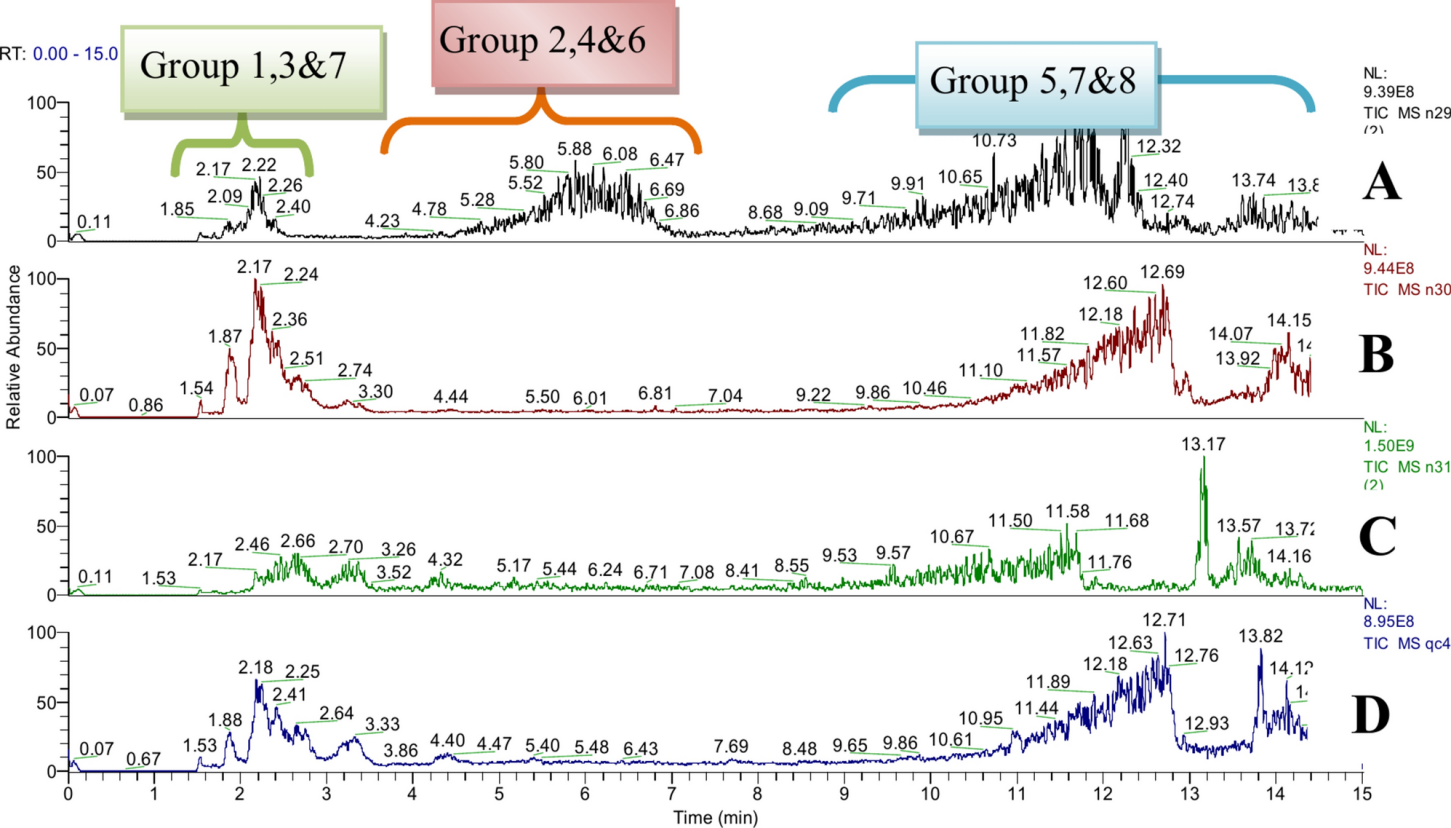
LC–MS/MS profiles of different groups of compounds, identified by tentative mass in successive extract fractionation (methanol (**A**); butanol (**B**); ethyl acetate (**C**) and QC sample (**D**)) of *Opuntia spp*.

**Fig. 3:**
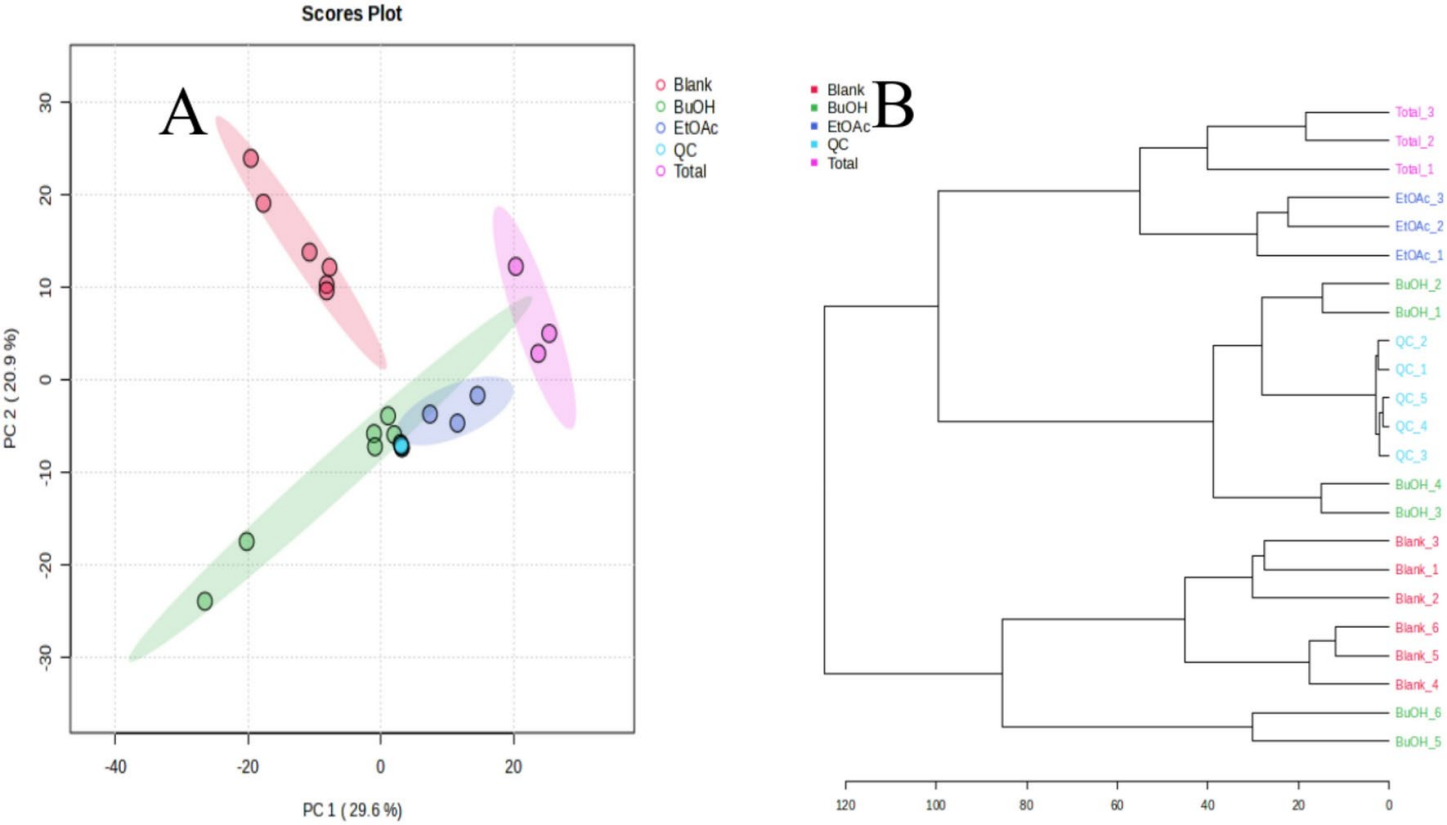
(**A**) Displays the positive mode correlations among different extracts of OFI-RF, as illustrated in the dendrogram. (**B**) Presents the PC1 loading plot for various extracts, which clusters the BuOH extracts in the same zone as the total extract. In contrast, the EtOAc fraction differs from both the total and blank fractions. This indicates that the fractionation process effectively distinguishes between different classes of compounds, leading to meaningful groupings^43^. Butanol = BuoH = BE, Ethylacetate = EtOAc = EE, and TT = Total extract.

**Fig. 4:**
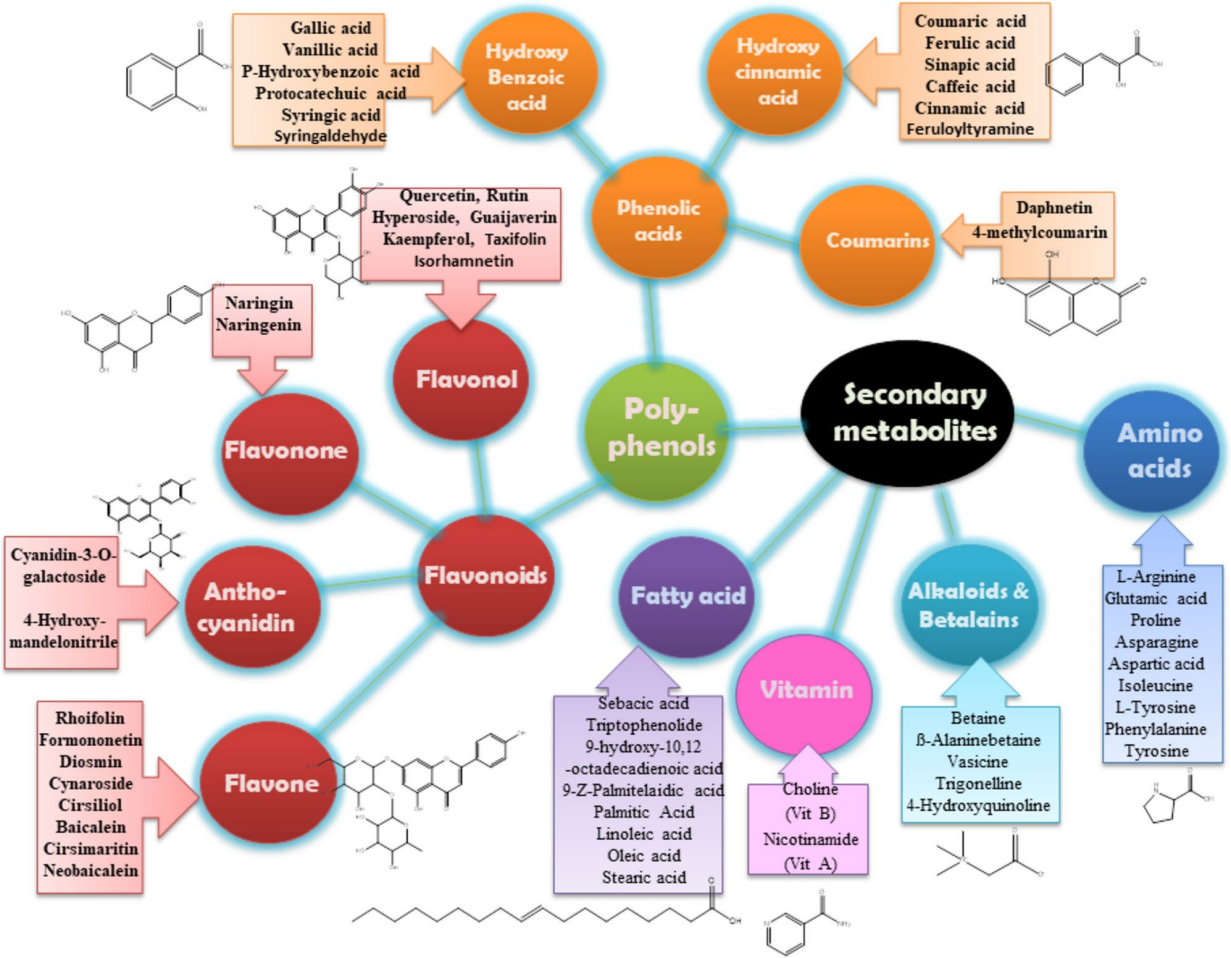
The OFI-RF extracts consist of a wide range of 101 essential metabolites that play a vital role as key elements in cellular biosynthetic pathways^44^. The identification of these essential metabolites was achieved through a comparison of mass spectra against the MS-Dial repository. Within the scope of this investigation, a compilation of diverse secondary metabolites was effectively determined via LC–MS analysis in Table 1.

For the structural elucidation of secondary metabolites, the extract was evaporated in a vacuum concentrator. During this procedure, the tube was placed in a tray rotating in a vacuum at room temperature. Dried extract samples were dissolved in methanol (1 mL) and subjected to solid-phase extraction (SPE) on silica gel RP/C18 (150 × 2.1 mm, 1.7 μm)^41^. The loaded SPE columns were filtrated by a syringe filter and then immediately subjected to the LC–MS analyses. The quantification of phenolic content through the Folin-Ciocalteu method does not provide a comprehensive assessment of the phenolic constituents present in extracts^22^. Therefore, LC/MS is the preferred methodology for both separating and quantifying polyphenolics (Figs. 2, 3 and 4).

Bioinformatics processing of LC/MS data resulted in the detection of 106,699 total signals in both modes (Table 1). Metabolites profiling via UPLC-HESI-MS/MS preliminary polyphenols were studied using ESI in negative and positive mode. The positive ionization mode resulted in a more detailed and intricate chromatographic profile compared to the negative mode. The Pearson correlation heat map of metabolic profiling revealed 68,362 total signals in the negative mode and 38,337 signals in the positive mode across various compound classifications (Fig. 3 and figures S1-5). The positive mode showed more intense signals for metabolite features that were consistent across all sampled communities. Specifically, in the negative mode, we identified 689 reference matches, 11,751 MS2 signals, and 23,782 suggested signals. In the positive mode, there were 573 reference matches, 12,584 MS2 signals, and 17,409 suggested signals. These results are illustrated by the base peak chromatogram (BPC) under the chosen analytical conditions and the chromatograms at 280 nm, which highlight intermediary molecules^42^.

**Table 1:** Mass spectral data for the tentative identification of polyphenolics rich and other components in *Opuntia* spp. in the different extract fractions.

No	RT [Min]	Metabolite identification	Chemical formula	[M-H]^-^			[M-H]^+^		∆ ppm		TE	EE	BE	References
				Measured & calculated	Fragmentation	∆ ppm	Measured & calculated	Fragmentation	UV ʎ_max_					
Group 1: Phenolic acids and Phenolic glycosides														
5	2.04	Quinic acid	C_6_H_8_O_7_	191.0188, 191.0186	173.0080, 129.0179, 111.0072, **85.0279**, 72.9914	1.0488	193.0344, 193.0343	175.0234[C_6_H_7_O_6_], 157.0133[C_6_H_5_O_5_], 129.0185[C_5_H_5_O_4_]	− 0.5180	206, 252	*	*	*	^7^
25	3.11	Gallic acid	C_7_H_6_O_5_	169.0132, 169.0131	136.9866, 125.0231, 107.0487, 97.0279, 65.0016	0.1100	171.0291, 171.0288	139.0028[C_6_H_3_O_4_], 125.0238[C_6_H_5_O_3_], 111.0082[C_5_H_3_O_3_]	1.9249	238, 284	*	*		
26	3.47	Cinnamic acid	C_9_H_8_O_2_	147.0440, 147.0441	119.0495[_87_]	− 0.3377	149.0599, 149.0597	121.0288[C_7_H_5_O_2_]	1.2389	209, 238	*	*		^7^
28	3.56	Benzoic acid + 2O, O-Hex;	C_13_H_16_O_9_	315.0717, 315.0711	165.0176[C_8_H_5_O_4_], 152.0101[C_7_H_4_O_4_], 108.0200[C_6_H_4_O_2_]	2.0880					*		*	
30	3.85	Piscidic acid derivative II	C_17_H_21_O_12_	417.1028, 417.1043	363.1712, 194.1855, 165.547, 138.2975, 111.0070	3.5962					*		*	^45^
31	3.86	Piscidic acid	C_11_H_12_O_7_	255.0508, 255.0499	211.0610, 179.0340, 165.0545, 149.0593, 107.0486, 72.9915	3.2213					*		*	^45^
32	3.88	Coumaric acid	C_9_H_8_O_3_	163.0387, 163.0390	119.0487[C_8_H_7_O]	− 1.6826	147.0442, 147.0441 [M + H− H2O] +	119.0495[C_8_H_7_O], 111.0444[C_6_H_7_O2], 91.0549	0.9075	241, 335	*	*	*	
33	3.91	Vanillic acid	C_8_H_8_O_4_	167.0337, 167.0339	137.0231, 123.0437, 93.0330, 65.0380	− 0.9776						*		
34	3.96	Protocatechuic acid	C_7_H_6_O_4_	153.0181, 153.0182	109.0280, 91.0172, 81.0329	− 1.2025	155.0340, 155.0339	137.0236[C_7_H_5_O_3_], 111.0446[C_6_H_7_O_2_]	1.0136	241, 320	*	*	*	^7^
40	4.68	*P*− Hydroxybenzoic acid	C_7_ H_6_ O_3_	137.0229, 137.0233	109.0278, 93.0329, 65.0380	− 2.9327	139.0390, 139.0390	111.0082[C_5_H_3_O_3_], 95.0498[C_6_H_7_O], 68.9980	0.2219	209, 238, 292				4
41	4.77	Benzoic acid	C_7_H_6_O_2_				123.0444, 123.0441	105.0342[C_7_H_5_O], 95.0498[C_6_H_7_O]	2.4486	241, 276	*	*	*	
44	4.97	Caffeoylquinic acid	C_16_H_18_O_9_	353.0870, 353.0871	191.0551[C_7_H_11_O_6_], 179.0334[C_9_H_7_O_4_] 173.0443[C_7_H_9_O_5_], 135.0435[C_8_H_7_O_2_]	0.2832					*	*		^7^
45	5.10	Trans-Caffeic acid	C_9_ H_8_ O_4_	179.0340, 179.0339	164.0108, 135.0437, 109.0278, 89.0229	0.4516	181.0495, 181.0501	163.0390[C_9_H_7_O_3_], 135.0443[C_8_H_7_O_2_], 107.0860	− 3.1422	202, 238	*			
47	5.20	Syringaldehyde	C_9_H_10_O_4_				183.0653, 183.0657	155.0704[C_8_H_11_O_3_], 123.0444[C_7_H_7_O_2_], 95.0498[C_6_H_7_O]	− 2.4944	209, 238	*			
49	5.37	Syringic acid	C_9_H_10_O_5_	197.0448, 197.0444	182.0212, 166.9977, 138.0308	1.6210	199.0597, 199.0601	181.0498[C_9_H_9_O_4_], 167.0388[C_8_H_7_O_4_], 140.0469[C_7_H_8_O_3_]	− 1.8941	238, 281	*		*	^7^
50	5.67	4-Methoxycinnamic acid	C_10_H_10_O_3_				179.0712, 179.0708	123.0444[C_7_H_7_O_2_], 111.0446[C_6_H_7_O_2_], 105.0341[C_7_H_5_O]	2.3686	271, 270	*	*	*	
53	5.89	3-O-Feruloylquinic acid	C_17_H_20_O_9_	367.10345, 367.10344	193.0496[C_10_H_9_O_4_], 129.0174[C_5_H_5_O_4_], 101.0229[C_4_H_5_O_3_]	− 0.0272				246, 275	*	*	*	
54	5.92	*P*-Coumaric acid	C_9_ H_8_ O_3_	163.0387, 163.0390	119.0487, 93.0331	− 1.4954						*		
56	6.13	Ferulic acid	C_10_H_10_O_4_	193.0497, 193.0495	178.0261, 149.0595, 134.0359, 121.0278	0.9213	195.0655, 195.0652	177.0547[C_10_H_9_O_3_], 133.0287[C_8_H_5_O_2_], 123..445[C_7_H_7_O_2_]	1.6432	238, 274	*	*	*	^7^
58	6.15	Sinapic acid	C_11_H_12_O_5_	223.0600, 223.0601	208.0375[C_10_H_8_O_5_], 164.0461[C_9_H_8_O_3_], 149.0236[C_8_H_5_O_3_]	− 0.3221	225.0758, 225.0757	207.0651[C_10_H_7_O_5_],	− 0.4442		*	*		
76	7.28	Vanillin	C_8_H_8_O_3_	151.0388, 151.0390	136.0153, 108.0203, 95.0123, 69.0704	− 0.8060	153.0547, 153.0546	125.0601[C_7_H_9_O_2_], 111.0441[C_6_H_7_O_2_], 93.0343[C6H5O]	0.6360	241, 267		*		
77	7.40	Feruloyltyramine	C_18_H_19_NO_4_	312.1237, 312.1230	190.0598[C_10_H_8_O_3_N], 178.0499[C_9_H_8_O_3_N]	1.9950	314.1393, 314.1387	175.0964[C_8_H_15_O_4_], 133.0865[C_6_H_13_O_6_]	1.8053	241, 263		*		
Group 2: Flavanols, Flavanonols , Flavonoids and Biflavonoids														
52	5.87	Rhoifolin	C_27_H_30_O_14_				579.1725, 579.1720	271.0597[C_15_H_11_O_5_]	− 0.8633	241, 271				
55	5.98	Isoquercetrin	C_21_H_20_O_12_				465.1037, 465.1030	303.0500[C_15_H_11_O_7_]	− 1.5050	241, 270	*	*	*	
57	6.14	Isorhamnetin 3-galactoside	C_22_H_22_O_12_	477.1044, 477.1028	357.0601[C_18_H_13_O_8_], 314.0432[C_16_H_10_O_7_], 285.0349[C_15_H_9_O_6_]	3.4546	479.1190, 479.1194	317.0659[C_16_H_13_O_7_]	0.8348	241, 270	*		*	
59	6.19	Diosmin	C_28_H_32_O_15_	607.2032, 607.2010	299.0199[C_15_H_7_O_7_], 270.0160[C_14_H_6_O_6_]	− 3.6231	609.1823, 609.1825	301.0707[C_16_H_13_O_6_]	0.3283	241, 270	*	*	*	
60	6.29	Cyanidin-3-O-galactoside	C_21_H_21_O_11_				449.1089, 449.1088	287.0550[C_15_H_11_O_6_], 177.1121[C_8_H_17_O_4_], 133.0862[C_6_H_13_O_3_]	− 0.2226	241, 270	*	*	*	
61	6.35	Kaempferol-3-O-rutinoside		593.1531, 593.1531	285.0401[C_15_H_9_O_6_]	4.0989					*		*	
63	6.42	Acacetin-7-O-neohesperidoside	C_28_H_32_O_14_				593.1878, 593.1871	356.0211[C_10_H_12_O_14_], 285.0753[C_16_H_13_O_5_]	− 1.1800	243, 271				
64	6.55	Naringin	C_27_H_32_O_14_	579.1741, 579.1762	549.1146[C_19_H_23_O_13_], 339.0713[C_15_H_15_O_9_], 271.0612[C_15_H_11_O_5_], 151.0023[C_7_H_3_O_4_]	3.6258				241, 270	*		*	^7^
65	6.65	Hyperoside	C_21_H_20_O_12_	463.0886, 463.0871	300.0273[C_15_H_8_O_7_], 271.0249[C_14_H_7_O_6_], 151.0019[C_7_H_3_O_4_]	3.2179							*	^7^
66	6.70	Tiliroside	C_30_H_26_O_13_	593.1300, 593.1320	447.0905[C_21_H_19_O_11_], 285.0403[C_15_H_9_O_6_], 151.0021[C_7_H_3_O_4_]	3.3719				241, 277	*	*		
67	6.74	Rutin	C_27_H_30_O_16_	609.1461, 609.1481	300.0715[C_16_H_13_O_6_], 151.0022[C_7_H_3_O_4_]	3.2832	611.1606, 611.1609	303.0492[C_15_H_11_O_7_]	0.4908	241, 277	*	*	*	^7^
68	6.79	Kaempferol 7-O-glucoside	C_21_H_20_O_11_	447.0931, 447.0922	284.0325[C_15_H_8_O_6_]	2.0608	449.1089, 449.1080	287.0550[C_15_H_11_O_6_]	− 2.0039	241, 270	*		*	
69	6.84	Guaijaverin	C_20_H_18_O_11_	433.0760, 433.0765	300.0275[C_15_H_8_O_7_], 271.0249[C_14_H_7_O_6_]	− 1.1266				241, 270	*	*	*	
70	6.91	Isorhamnetin-3-O-rutinoside (Narcissin)	C_28_H_32_O_16_	623.1550, 623.1561	315.0510[C_16_H_11_O_7_], 271.0247[C_14_H_7_O_6_], 151.0027[C_7_H_3_O_4_]	1.6207	625.1766, 625.1760	317.0656[C_16_H_13_O_7_]	− 0.9597	241, 267			*	^46^
71	6.94	Isorhoifolin	C_27_H_30_O_14_	577.1617, 577.1615	269.0454[C_15_H_9_O_5_]	− 0.3465				241, 270	*	*		
72	6.96	Cynaroside	C_21_H_20_O_11_	447.0948, 447.0922	285.0400[C_15_H_9_O_6_], 169.0132[C_7_H_5_O_5_], 111.0074[C_5_H_3_O_3_]	5.8149					*	*		^7^
73	7.08	Isorhamnetin 3-galactoside	C_22_H_22_O_12_	477.1044, 477.1028	357.0601[C_18_H_13_O_8_], 314.0432[C_16_H_10_O_7_], 285.0349[C_15_H_9_O_6_]	3.4546				241, 270	*		*	
74	7.16	Dihydrokaempferol	C_15_H_12_O_6_	287.0562, 287.0550	259.0614[C_14_H_11_O_5_], 243.0663[C_14_H_11_O_4_], 125.0231[C_6_H_5_O_3_]	4.0700					*		*	^46^
75	6.96	Cynaroside	C_21_H_20_O_11_	447.0948, 447.0922	285.0400[C_15_H_9_O_6_], 169.0132[C_7_H_5_O_5_], 111.0074[C_5_H_3_O_3_]	5.8149					*		*	^7^
77	8.26	Quercetin	C_15_H_10_O_7_	301.0352, 301.0343	178.9974[C_8_H_3_O_5_], 151.0024[C_7_H_3_O_4_], 121.0280[C_7_H_5_O_2_]	3.2180	303.0501, 303.0499	285.0394[C_15_H_9_O_6_], 229.0503[C_13_H_9_O_4_], 177.0547[C_10_H_9_O_3_] 153.0184[C_7_H_5_O_4_]	0.6971	241, 270	*		*	^7^
79	8.39	Naringenin	C_15_H_12_O_5_	271.0611, 271.0601	151.0022[C_7_H_3_O_4_]	3.5628	273.0757, 273.0757	153.0184[C_7_H_5_O_4_]	− 0.1314	241, 270	*		*	^7^
80	8.48	Formononetin	C_16_H_12_O_4_				269.0804, 269.0808	254.0563[C_15_H_10_O_4_]	− 1.5667	245	*			
81	8.77	Baicalein	C_15_H_10_O_5_	269.0456, 269.0444	255.0544[C_14_H_6_O_3_], 165.0546[C_9_H_9_O_3_]	3.0021	271.0602, 271.0601	257.05645[C14H8O3], 165.0702[C9H9O3]	0.2978	245, 270	*	*	*	
82	8.88	Jaceidin *flavonoid*	C_18_H_16_O_8_	359.0776, 359.0761	344.0536[C_17_H_12_O_8_], 329.0298[C_16_H_9_O_8_], 301.0359[C_15_H_9_O_7_]	4.1575				241, 277		*	*	
83	9.04	Cirsiliol	C_17_H_14_O_7_	329.0669, 329.0667	314.0423[C_16_H_10_O_7_], 199.1327	4.0160				245, 270	*	*	*	^7^
84	9.18	Kaempferol *Flavonols*	C_15_H_10_O_6_	285.0404, 285.0394	151.0021[C_8_H_7_O_3_]	3.6514	287.0550, 287.0550	151.0392[C_8_H_7_O_3_]	− .1824		*	*	*	^46^
85	9.19	Kaempferol 3-O-methyl ether	C_16_H_12_O_6_	299.0551, 299.0550	284.0322[C_15_H_8_O_6_], 255.0287 [C_14_H_6_O_3_] 227.0348[C_13_H_7_O_4_]	0.4372					*		*	^46^
86	9.25	Isokaempferide	C_16_H_12_O_6_	299.0561, 299.0550	284.0324[C_15_H_8_O_6_], 255.0298[C_14_H_7_O_4_], 227.0341[C_13_H_7_O_4_]	3.6006					*		*	
87	9.23	Taxifolin	C_15_H_12_O_7_	303.0662, 303.0652	285.0555[C_15_H_10_O_6_]	3.3237				245, 270	*		*	^46^
88	9.52	Cirsimaritin	C_17_H_14_O_6_	313.0714, 313.0707	298.0480[C_16_H_10_O_6_], 283.0250[C_15_H_7_O_6_]	2.3846					*		*	MSDial
89	9.53	Isorhamnetin	C_16_H_12_O_7_	315.1808, 315.1802	300.0290[C_15_H_8_O_7_], 187.1332[C_10_H_19_O_3_]	1.7152	317.0655, 317.0656	302.0859[C_15_H_10_O_7_],	− 0.2790	245, 270	*	*	*	^47^
90	9.86	5-hydroxy-6,7-dimethoxyflavone	C_17_H_14_O_5_				299.0910, 299.0914	195.0288[C_9_H_7_O_5_], 137.0600[C_8_H_9_O_2_]	− 1.2241		*	*		
92	9.94	Skullcapflavone II Neobaicalein	C_19_H_18_O_8_	373.0906, 373.0918	358.0698[C_18_H_14_O_8_]	− 3.1821				245, 270	*			
94	11.15	Acacetin	C_16_H_12_O_5_	283.0611, 283.0601	268.0374[C_15_H_5_O_5_]	3.1596					*			^7^
Group 3: Alkaloids and Pyridine carboxylic acids*														
24	2.81	Vasicine	C_11_H_12_N_2_O				189.1024, 189.1028	101.0749[C_4_H_9_ON_2_], 69.0344[C_4_H_5_O]	− 1.8812	213, 227		*		
29	3.80	Trigonelline	C_7_H_7_NO_2_	136.0389, 136.0393	108.0439, 192.0489, 66.0333	− 2.7919	138.0550, 138.0550	110.0605[C_6_H_8_ON], 95.0498[C_6_H_7_O]	0.4937	209, 238, 292		*		
27	3.60	4-Hydroxymandelonitrile	C_8_H_7_NO_2_	148.0389, 148.0393	120.0438[C_7_H_6_ON], 92.0495[C_6_H_6_N]	− 2.5656	150.0551, 150.0550	122.0603[C_7_H_8_ON], 94.0658[C_6_H_8_N]	0.8609	202, 234, 277		*		
20	2.38	Isonicotinic acid;	C_6_H_5_NO_2_				124.0396, 124.0393	96.0450[C_5_H_6_NO], 80.0502[C_5_H_6_N]	2.7197	216		*		
35	4.09	Citrazinic acid	C_6_ H_5_ O_4_ N	154.0131, 154.0135	133.9879, 110.0232, 66.0333	− 2.4467					*	*	*	
38	4.61	4-Hydroxyquinoline	C_9_H_7_NO	144.0441, 144.0444	135.419, 116.0491, 68.5640	− 2.1364	146.0602, 146.0600	118.0655[C_8_H_8_N]	1.0645	209, 234, 277	*	*	*	
Group 4: Amino acids and Betalains Derivatives														
1	1.59	Betaine	C_5_H_11_NO_2_				118.0866, 118.0863	77.0393	3.1247	202, 224	*			
3	1.97	L-Arginine	C_6_H_14_N_4_O_2_				175.1191, 175.1190	158.0926[C_6_H_12_O_2_N_3_], 130.0978[C_5_H_12_ON_3_], 116.0711[C_5_H_10_O_2_N]	0.9886	227, 284	*			
6	2.08	Glutamic acid	C_5_H_9_NO_4_				148.0603, 148.0604	130.0501[C_5_H_8_O_3_N], 102.0555[C_4_H_8_O_2_N], 88.0400[C_3_H_6_O_2_N], 84.0452[C_4_H_6_ON]	− 0.8886	202, 213, 281	*	*		^10^
7	2.11	Proline	C_5_H_9_NO_2_				116.0710, 116.0706	70.0660[C_4_H_8_N]	3.1319	202, 224	*	*		^10^
8	^©^2.17	L-beta-Homoproline	C_6_H_13_NO_2_				130.0866, 130.0863	84.0816[C_5_H_10_N], 70.0661[C_4_H_8_N]	2.3673	216	*	*		
10	2.20	Asparagine	C_4_H_8_N_2_O_3_				133.0611, 133.0608	116.0347[C_4_H_6_NO_3_], 87.1005[C_4_H_11_N_2_], 74.0245[C_2_H_4_O_2_N]	2.6908		*	*		
11	2.20	Aspartic acid	C_4_H_7_NO_4_				134.0450, 134.0448	116.0347[C_4_H_6_NO_3_], 88.0401[C_3_H_6_O_2_N], 74.0245[C_2_H_4_O_2_N]	1.7098		*	*	*	
14	2.26	Beta-Alaninebetaine	C_6_H_13_NO_2_	130.0858, 130.0863	85.0281[C_4_H_5_O_2_]	− 3.6149					*	*	*	
16	2.33	Isoleucine	C_6_H_13_NO_2_				132.1021, 132.1019	86.0972[C_5_H_12_N], [69.0708]	1.7949		*	*		
18	2.32	L-TYROSINE	C_9_H_11_NO_3_				182.0813, 182.0817	165.0584[C_9_H_9_O_3_], 147.0441[C_9_H_7_O_2_], 136.0759[C_9_H_10_ON], 123.0445[C_7_H_7_O_2_]	− 2.2192	213, 227, 252	*	*		^10^
22	2.44	L-5-Oxoproline	C_5_H_7_NO_3_				130.0502, 130.0499	102.0556[C_4_H8O_2_N], 84.0452[C_4_H_6_NO]	2.4338	213, 220	*	*		^10^
23	2.73	Phenylalanine	C_9_H_11_NO_2_				166.0863, 166.0863	149.0597[C_9_H_9_O_2_], 120.0812[C_8_H_10_N]	0.2923	224, 317	*	*		^10^
39	4.63	Tyrosine	C_9_H_11_NO_3_	180.0653, 180.0655		− 1.1778	182.0813, 182.0812	147.0441[C_9_H_7_O_2_] 136.0759[C_8_H_10_NO]	0.7927	213, 227	*			^10^
Group 5: Carboxylic acids														
4	1.99	4-Hydroxyphenyllactic acid	C_9_H_10_O_4_	181.0496, 181.0495	166.0261[C_8_H_6_O_4_]	0.5610								
9	2.18	Shikimic Acid	C_7_H_10_O_5_	173.0440, 173.0444	155.0336[C_7_H_7_O_4_], 137.0230[C_7_H_5_O_3_], 93.0330[C_6_H_5_O]	− 0.1822	175.0602, 175.0601	157.0131[C_6_H_5_O_5_], 139.0027[C_6_H_3_O_4_], 111.0081[C_5_H_3_O_3_]	0.3740	220, 252	*	*	*	
17	2.31	Malic acid^a^	C_4_ H_6_O_5_	133.0127, 133.0131	115.0021, 89.0227, 71.0121	− 3.1871					*	*		4
21	2.82	Citric acid^a^	C_6_ H_8_ O_7_	191.0188, 191.0186	173.0080, 145.9975, 129.0179, 111.0072, **87.0071**	0.8091					*	*		4
19	2.36	Mucic acid	C_6_H_10_O_8_|	209.0293, 209.0292	191.0189[C_6_H_7_O_7_], 133.0129[C_5_H_7_O_5_]	04,937					*	*		
43	4.92	Kynurenic acid	C_10_H_7_NO_3_	188.0345, 188.0342	159.0316, 144.0441, 129.0535, 73.0925	1.7354	190.0499, 190.0504	162.0550[C_9_H_8_O_2_N]	− 2.5047	241, 270	*	*		
46	5.14	3-phenyllactic acid	C_9_H_10_O_3_	165.0545, 165.0546	147.0438, 119.0488, 72.9915	− 0.6120					*	*		
62	6.36	Azelaic acid	C_9_H_16_O_4_	187.0965, 187.0965	169.0858, 143.1057, 125.0957, 111.0073, 97.0641, 81.0328, 67.0172	0.0595					*	*		
Group 6: Coumarins														
36	4.42	4-methylcoumarin	C_10_H_8_O_2_				161.0599, 161.0597	133.0650[C_9_H_9_O]	0.9572		*	*		
42	4.88	Daphnetin	C_9_H_6_O_4_	177.0184, 177.0182	133.0278, 105.0329, 85.0279	− 0.2637					*		*	
Group 7: Vitamins and organic *compounds*														
2	1.95	Choline (Vit B)	C_**5**_H_14_NO				104.1075, 104.1070	60.0818[C_3_H_10_N]	5.11684	206, 284	*	*	*	
12	^©^2.22	Nicotinamide (Vit B3)	C_**6**_H_**6**_N_**2**_O				123.0555, 123.0553	96.0498[C_6_H_7_O], 80.0502[C_5_H_6_N]	1.8046	206, 234	*	*	*	
13	2.28	Sucrose	C_**12**_H_**22**_O_**11**_	341.1086, 341.1078	237.0616[C_8_H_13_O_8_], 179.0549[C_6_H_11_O_6_], 143..0336[C_6_H_7_O_4_]	2.1803					*		*	MSdial
15	2.29	Adenosine	C_**10**_H_**13**_N_**5**_O_**4**_				268.1035, 268.1040	136.0619[C_5_H_6_N_5_]	− 2.1478	202, 213,	*			^48^
37	4.56	4-Hydroxybenzoylcholine	C_**12**_H_**18**_NO_**3**_				224.1281, 224.1281	165.0545[C_9_H_9_O_3_]	0.1043		*		*	
48	5.35	Thymol	C_**10**_H_**14**_O				151.1119, 151.1117	133.1015[C_10_H_13_], 109.0654[C_7_H_9_O], 93.0706[C_7_H_9_]	1.2015	241, 270	*	*	*	
51	^©^5.82	Loliolide	C_**11**_H_**16**_O_**3**_				197.1172, 197.1172	179.1068[C_11_H_15_O_2_], 161.0958[C_11_H_13_O], 141.0548[C_7_H_9_O_3_]	0.0627	209, 238, 274	*		*	
78	8.38	2-Mercaptobenzothiazole	C_**7**_ H_**5**_ N S_**2**_	165.9780, 165.9780	134.0057, 116.9270, 79.8595	0.1790					*		*	
93	10.48	N-Butylbenzenesulfonamide	C_**10**_H_**15**_NO_**2**_S				214.0896, 214.0891	158.0257[C_6_H_8_NO_2_S], 141.0002[C_6_H_5_O_2_S]	− 2.6890	250, 341	*			
Group 8: Fatty acid profiles														
77	7.45	Sebacic acid	C_**10**_H_**18**_O_**4**_	201.1124, 201.1121	183.1016[C_10_H_15_O3], 139.1114[C_9_H_15_O]	1.4482					*	*		
91	9.92	Triptophenolide	C_**20**_H_**24**_O_**3**_	311.1655, 311.1642	267.1965	4.1625				245, 270	*	*		MSDial
95	11.82	FA 18:1 + 3O;	C_**18**_H_**34**_O_**5**_	329.2335, 329.2323	229.1442[C_12_H_21_O_4_], 211.1333[C_12_H_19_O_3_]	3.6718				245, 270	*	*		
96	12.50	9-hydroxy-10,12-octadecadienoic acid	C_**18**_H_**32**_O_**3**_	295.2277, 295.2268	277.2168[C_18_H_29_O_2_], 249.2224[C_17_H_29_O], 141.1271[C_9_H_17_O]	3.0140					*	*		
97	13.17	9-Trans-Palmitelaidic acid	C_**11**_H_**12**_O_**7**_	253.2160, 253.2162	180.1960	−0.6822					*	*		^49^
98	13.34	Palmitic Acid	C_**16**_H_**32**_O_**2**_	255.2326, 255.2319	191.1146	2.8121					*	*		^49^
99	13.44	Linoleic acid	C_**18**_H_**32**_O_**2**_	279.2330, 279.2319	261.2229[C_18_H_29_O]	4.1001					*	*		^49^
100	14.16	Oleic acid	C_**18**_H_**34**_O_**2**_	281.2487, 281.2475	263.2391[C_18_H_31_O]	4.1990					*	*		^49^
101	14.51	Stearic acid	C_**18**_H_**36**_O_**2**_	283.2646, 283.2632	209[C_9_H_5_O_6_]	4.9425					*	*		^49^

Methanol Extract; Total Extract, TE; Butanol Extract, BE; Ethylacetate Extract, EE.

Significant values are in bold.

*Indicates compounds that were identified in this fraction.

### Pharmacological study

#### In vitro study

##### In vitro antidiabetic activity

(i) Amylase inhibition

(ii) Glucosidase inhibition

**Fig. 5:**
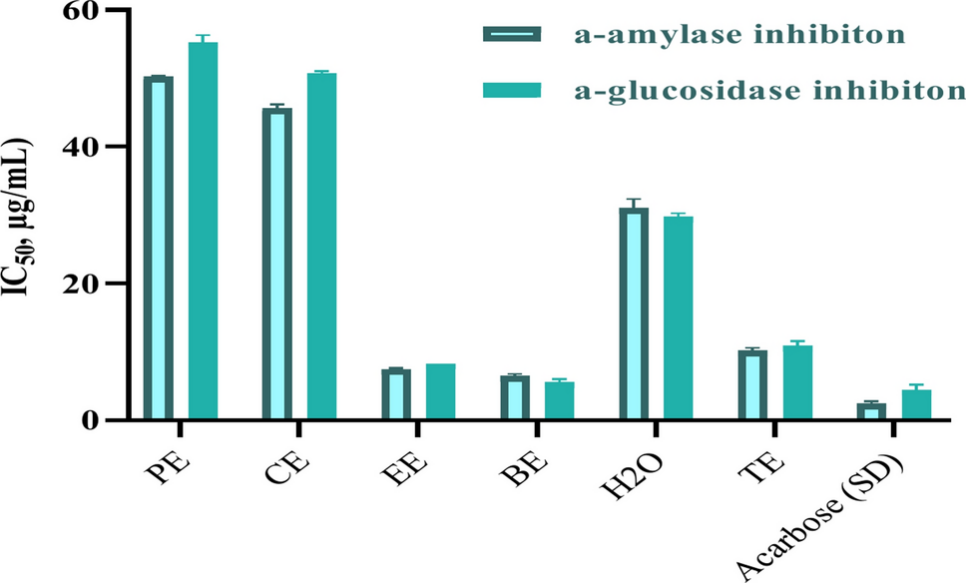
In vitro antidiabetic of amylase and glucosidase enzymes.

The highest levels of α-amylase and α-glucosidase enzymes activity were detected in the butanol and ethylacetate fractions, with respective IC_50_ values of 6.51 ± 0.24 and 7.51 ± 0.17, and by IC_50_ values of 5.58 ± 0.45 and 8.33 ± 0.02, respectively. This observation aligns with the outcomes of total phenolic and total flavonoids analyses, as well as the LC/MS quantification results, as illustrated in Fig. 5 and Table S1. The mean ± standard error of the mean (s.e.m) was determined using t-test analysis by GraphPad Prism software (USA, version 8.0.1). The level of statistical significance was adjusted to *p*-value of less than 0.05.

##### In vitro antioxidant activity

**Fig. 6:**
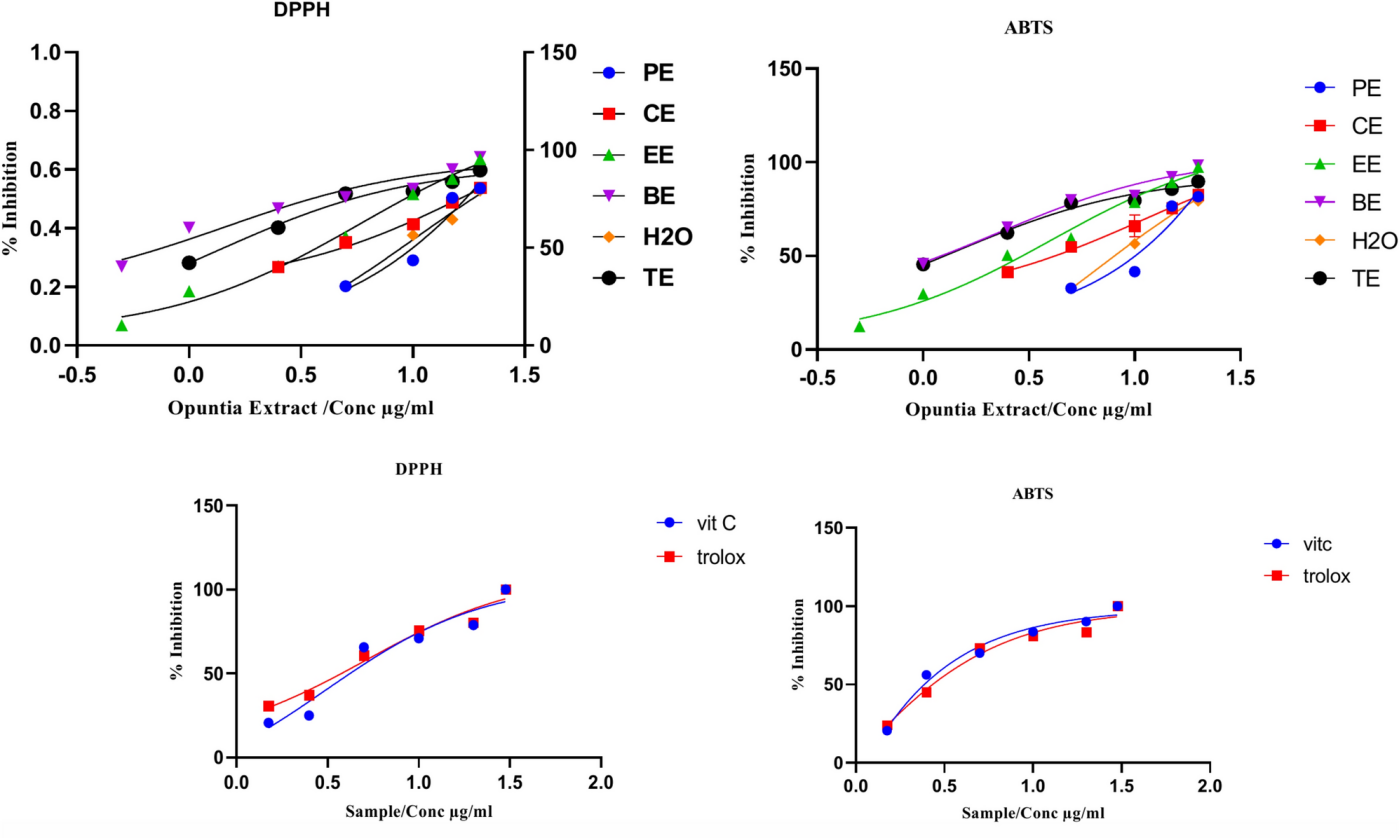
Concentration–response curve for the absorbance at 734 nm for ABTS^•+^ as a function of concentration of standard Trolox solution. (Six separately prepared stock standard solutions).

(i) DPPH^.^ assay method: Findings of the antioxidant activity were obtained by conducting DPPH assay for total methanol (TE), petroleum ether fraction (PE), dichloromethane fraction (CE), ethylacetate fraction (EE), butanol fraction (BE) and water fraction (H_2_O) as shown in Fig. 6 and Table S2. All extracts were evaluated against vitamin C and trolox as reference standards^50^. Results revealed a descending order of IC_50_ values activity as follows: 48.23, 18.74, 10.20, 5.78, 1.44, and 1.35 µg/ml for PE > CE > H_2_0 > EE > BE and TE, respectively).

(ii) ABTS^•+^ radical cation: Data of the current study provide a quantification of the antioxidative efficacy against ABTS^**•**+^ radical formation. Assessment of reduction in the radical cation was determined as a percentage decrease in absorbance at 734 nm. In Table S3, the duration of the inhibitory action of trolox and ascorbic acid on ABTS^•+^ radical cation absorbance (at 734 nm) was depicted as the conventional reference substances. A comparison was shown between the values of the two standards and those of TE, PE, CE, EE, BE, and H_2_O^51^.

The antioxidant activity was directly proportional to concentration. This finding was evident in both standards, as well as all six specimens under investigation. Values of IC_50_ were arranged in descending order, in the following sequence: 120.50, 10.89, 10.20, 6.93, 4.02, and 1.43 µg/ml for PE > CE > H20 > EE > BE and TE, respectively (Fig. 6 and Table S3).

Furthermore, the TE, BE, and EE extracts exhibited the strongest antioxidant activity compared to all other extracts and standard compounds, as they effectively inhibited DPPH and ABTS in Fig. 6.

#### In vivo* study*

##### Metabolic changes

**Fig. 7:**
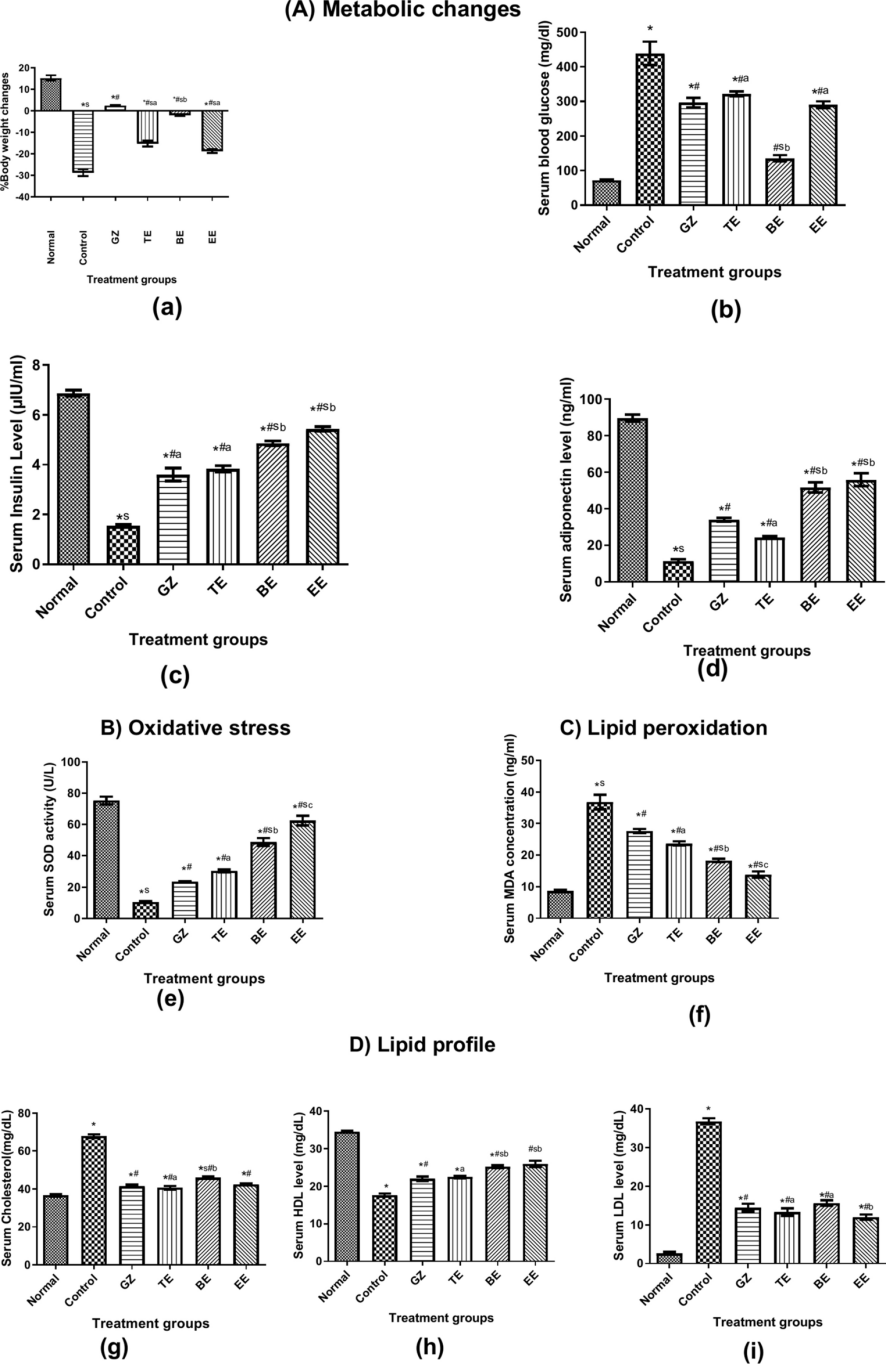
Effect of daily oral treatment with GZ, TE, BE and EE for 3 successive weeks on *A) metabolic changes*: % weight changes (**a**), serum glucose (**b**), serum insulin (**c**) and adiponectin (**d**), *B) Oxidative stress*: SOD activity (**e**), *C) Lipid peroxidation*: MDA (**f**), *C) Lipid Profile*: cholesterol (**g**), HDL (**h**) LDL (**i**) in normal and diabetic rats (n = 8). Each value represents mean ± sem. * vs. normal, ^#^ vs. control, ^s^ vs. GZ reference standard, values not sharing the same alphabet differ significantly at *p* < 0.05. (Gliclazide = GZ, methanol fraction = TE, butanol fraction = BE, and ethylacetate fraction = EE).

*% Body weight changes*: Fig. 7a illustrated that normal animals showed a mean of 15% weight change at the end of the 3 week study period; however STZ induced a significant threefold reduction as compared to normal group. Oral administration of GZ, BE or EE extracts significantly elevated the % weight change by 31.3 and 26.8 and 10%, respectively as compared to diseased control animals; however this change remained significantly lower in comparison with the normal value. Noteworthy, BE extract exhibited significant improvement superior to that of the TE and EE extracts at *p* < 0.05.

*Estimation of serum glucose*: STZ significantly raised serum glucose level to 6 folds with respect to normal value as shown in Fig. 7b. GZ daily administration, as reference standard for 3 weeks, revealed a significant reduction in blood glucose level to 67.4% that of the diabetic group. Likewise, daily oral treatment with TE and EE extracts exhibited a marked reduction to 73 and 66% that of the diseased rats. Noteworthy, oral treatment with BE caused a marked improvement by 69 and 54% as compared to that of diabetic and GZ groups, respectively, almost approaching near normal value at *p* < 0.05, therefore showing better effect than the other two extracts.

*Estimation of serum insulin*: As shown in Fig. 7c, STZ significantly depressed insulin level to 23% that of the normal value. Daily treatment of diabetic rats with GZ, TE, BE and EE for 3 weeks exhibited significant rise to 2.2, 2.4, 3 and 3.4 folds that of control untreated animals, respectively, where BE and EE showed superior effect to GZ.

*Estimation of serum adiponectin*: Untreated diabetic rats exhibited a sharp depression in serum adiponectin level to 13% that of the normal group as illustrated in Fig. 7d. Daily oral treatment with GZ, TE, BE and EE for 3 weeks significantly elevated its level to 3, 2, 4.5 and 5 folds that of the untreated rats, respectively, but was still significantly lower than normal value. Interestingly, BE and EE showed better effect than that observed in TE group at *p* < 0.05.

##### Antioxidant effect

*Estimation of Serum SOD*: According to Fig. 7e, STZ induced a marked reduction in enzyme activity to 14% that of the normal value. Daily oral administration of GZ, TE, BE and EE significantly raised SOD activity to 2.2, 2.8, 4.6 and 6 folds that of the diseased rats, respectively, but was still remarkably lower than normal value. Again, BE and EE showed superior improvement in enzyme activity to 2 and 2.7 folds that of the GZ group, the latter of which was best of all extracts at *p* < 0.05.

##### Effect on lipid peroxidation

*Estimation of serum MDA:* Lipid peroxidation was estimated as MDA concentration in serum (Fig. 7f). STZ significantly raised its level to 4 folds the normal value. Interestingly, treatment of diabetic animals with GZ, TE, BE and EE significantly reduced MDA level to 75, 64, 50 and 38% the value of the diseased group respectively, however they remained significantly higher than that of normal animals. Noteworthy, EE showed better effect in comparison with standard and other extracts at *p* < 0.05.

##### Effect on lipid profile

*Estimation of serum cholesterol*: According to Fig. 7g, streptozotocin-injected animals revealed a 1.8 fold elevation in serum cholesterol level with respect to normal value. Daily oral intake of GZ, TE, BE and EE extracts for 3 successive weeks markedly decreased the value to approximately 62, 60, 68 and 62% that of the diabetic group, respectively at *p* < 0.05.

*Estimation of serum HDL*: Fig. 7h showed that STZ injection significantly lowered serum HDL value to almost half that of normal animals, however daily treatment with GZ, TE, BE and EE induced a significant rise by 22, 28, 39, 44%, respectively in comparison with that of the untreated group, with superior effect revealed by BE and EE over the group treated with GZ as reference drug at *p* < 0.05.

*Estimation of serum LDL*: Untreated diabetic rats exhibited an 11 fold elevation in serum LDL with respect to normal animals (Fig. 7i). Oral treatment with GZ, TE, BE and EE markedly reduced values to 39, 36, 42 and 33% that of the diseased group, respectively, hence superior effect was noticeable in EE-treated group at *p* < 0.05.

### Histopathological study

**Fig. 8:**
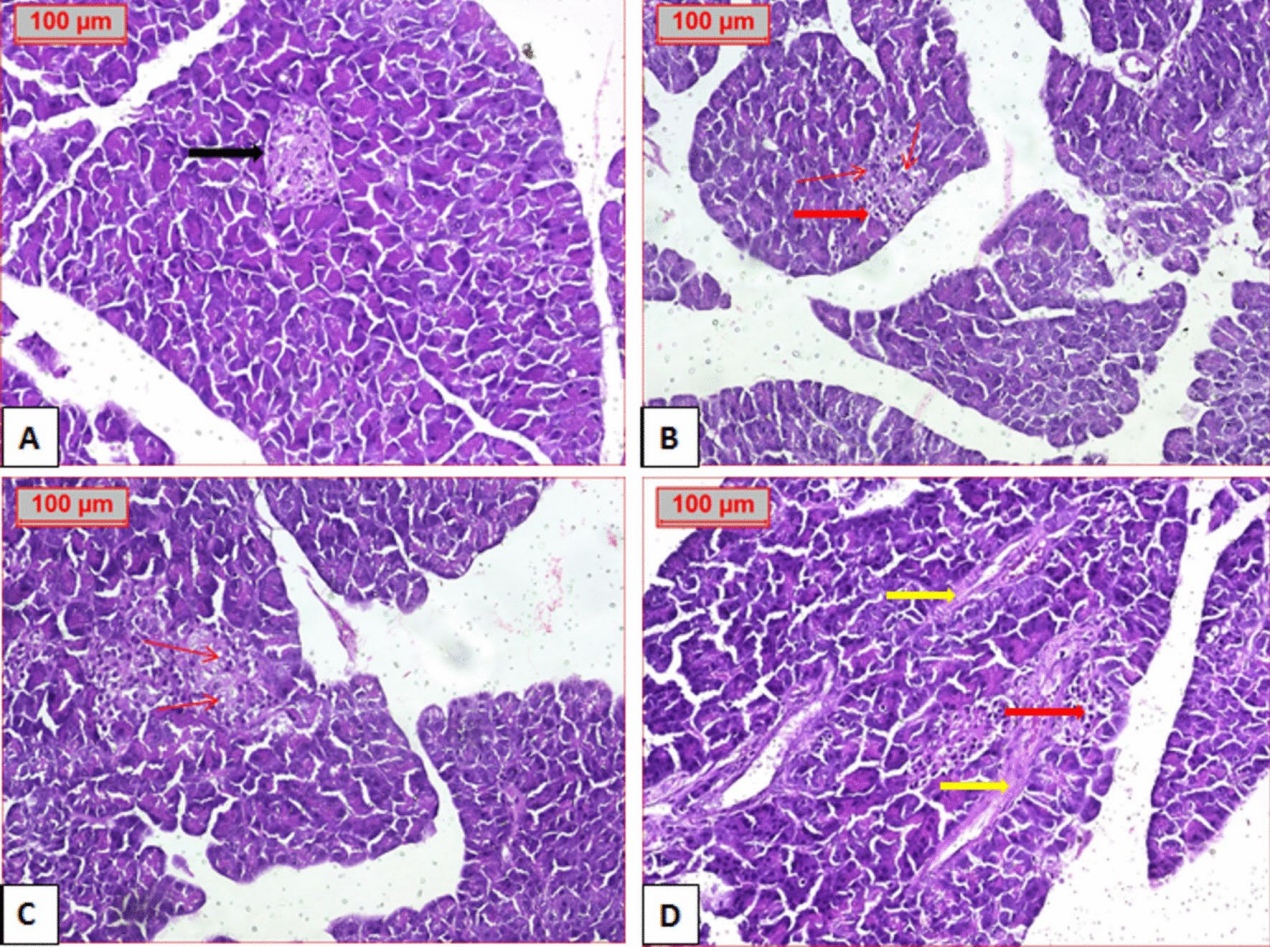
Photomicrograph of pancreatic tissue. (**A**) Normal group showing normal structure of pancreatic tissue and islet of Langerhans (black arrow). (**B**, **C**, **D**) Diabetic pancreas showing decreased cell mass (thick red arrow) and increased vacuolation (thin arrow). Perivascular fibrosis (yellow arrow) (H&E stained, × 20).

As illustrated in Fig. 8 histopathological photomicrograph of the pancreas in normal group showed normal acini, with normal cellularity in the islets of Langerhans (Fig. 8A). Diabetic control group showed extensive damage to islets of Langerhans with disappearance of its borders. There is also a decrease in number of islet cells with permanent vesicular nuclei and appearance of vacuolation and perivesicular fibrosis (Fig. 8B–D).

**Fig. 9:**
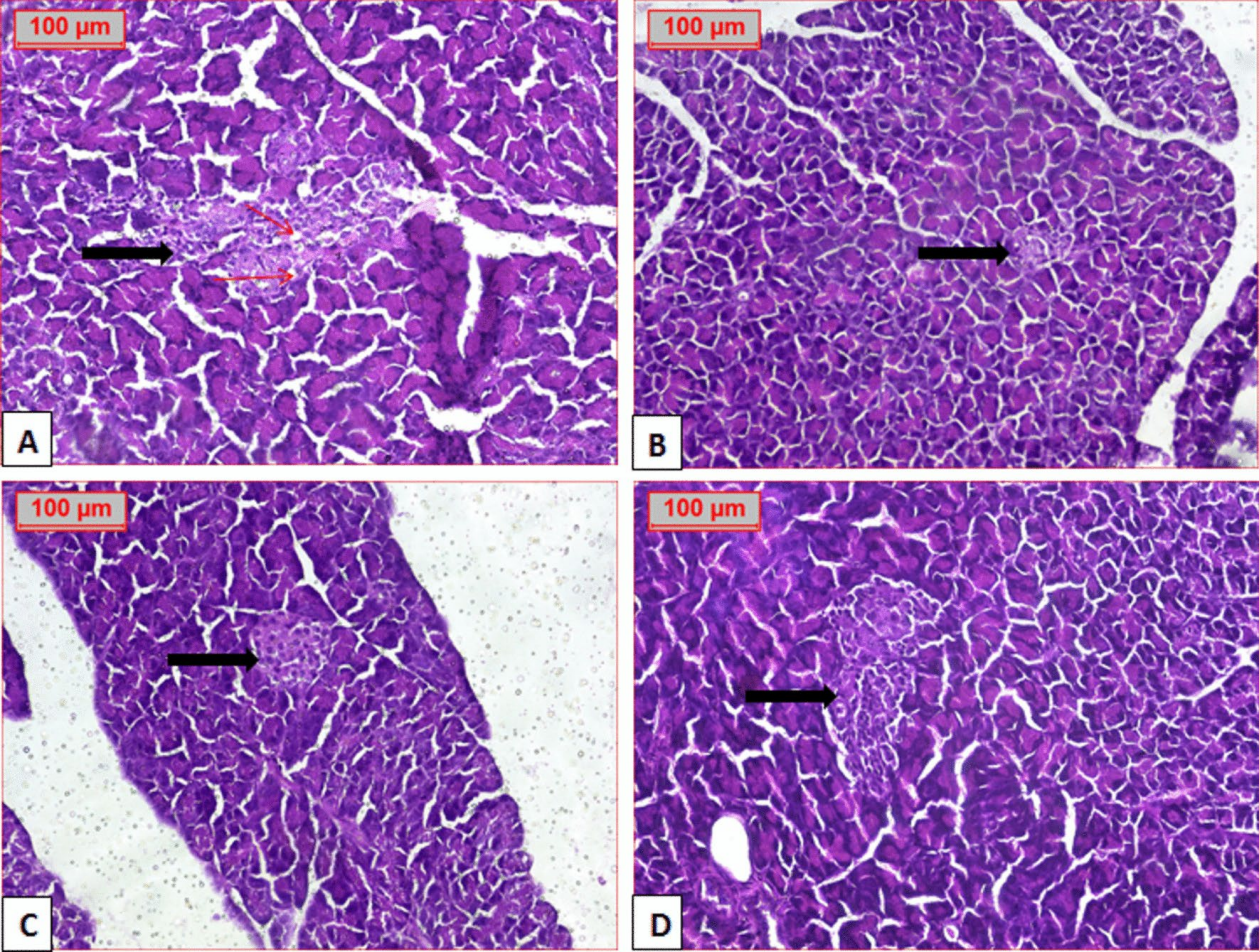
Photomicrograph of treated diabetic pancreas; (**A**) GZ group showing normal structure of pancreatic tissue (black arrow) with vacuolation (thin arrow), (**B**) TE group show normal pancreatic islet, (**C**) BE group show normal pancreatic islet and (**D**), EE group show normal pancreatic islet (H&E stained, × 20).(Gliclazide = GZ, methanol fraction = TE, butanol fraction = BE, and ethylacetate fraction = EE).

Treatment of diabetic group with GZ showed improvement of pancreatic islets with mild vacuolation. TE-treated group showed normal but smaller appearance in islet size. BE group revealed normal appearance with improved islet size. While administration of EE preserved normal appearance of pancreatic islet (Fig. 9).

These results indicated that treatment with selected extracts could repair islet damage and improve the structural integrity of pancreatic islet beta cells and tissues with variable degree, where marked improvement was observed from the extracts in the following order EE > BE > TE (Fig. 9).

**Fig. 10:**
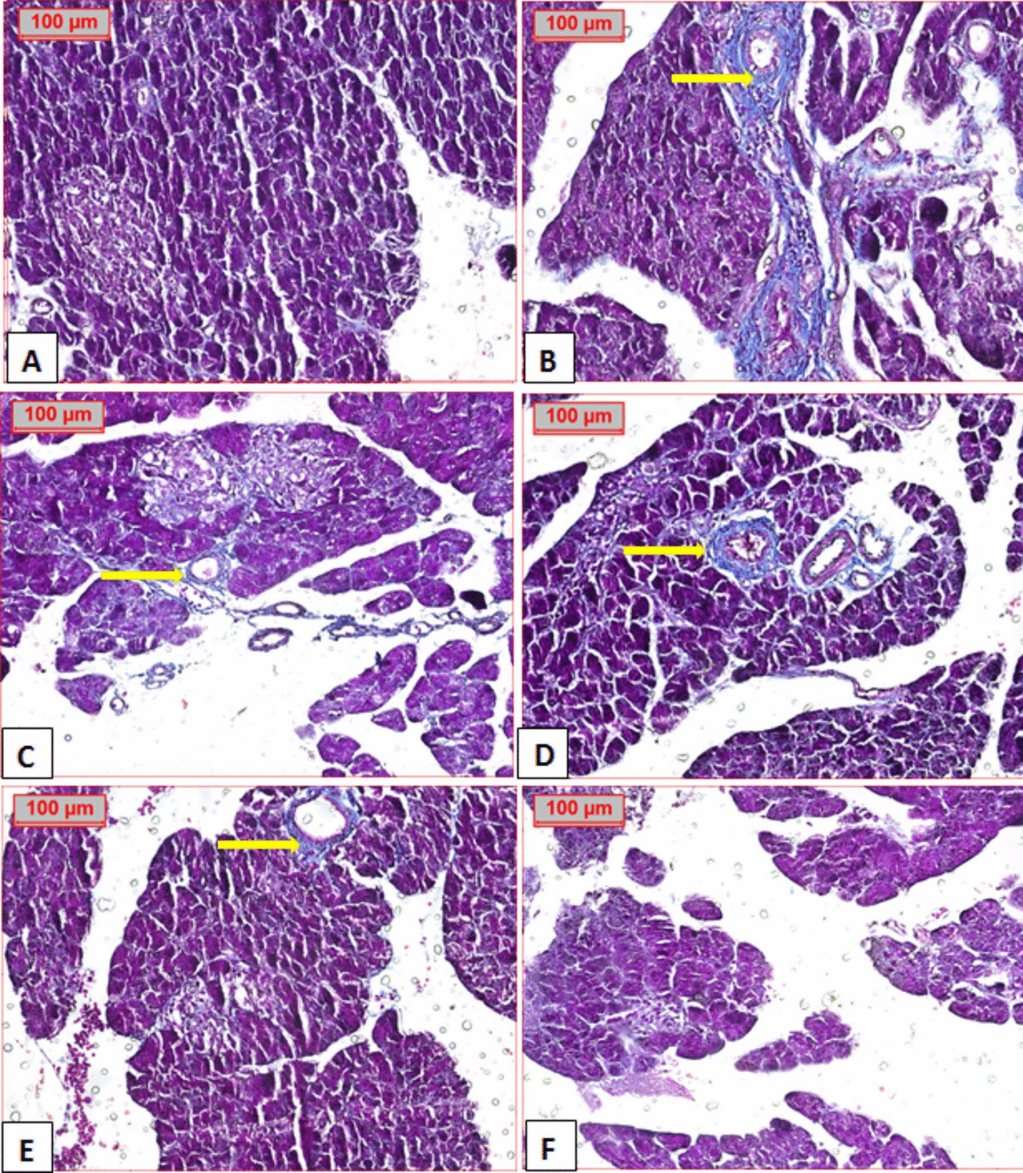
Photomicrograph show perivascular fibrosis (arrow) in different groups. (**A**) normal group, (**B**) diabetic group, (**C**) GZ group, (**D**) TE group, (**E**) BE group and (**F**) EE group. (Masson stained, × 20).

**Fig. 11:**
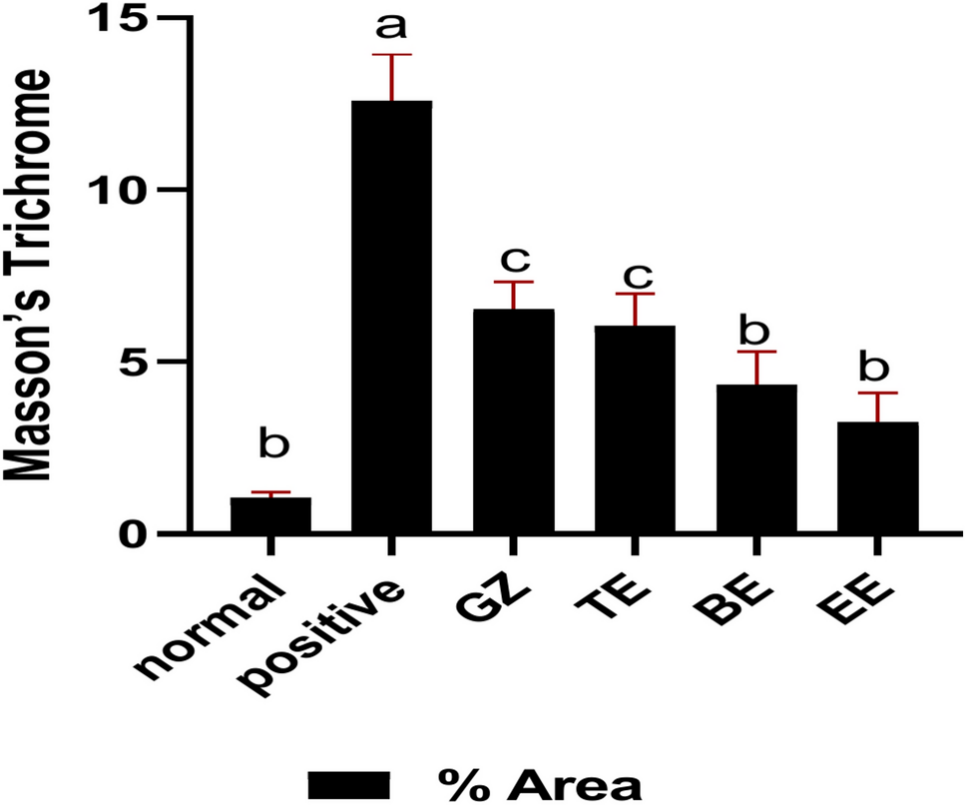
Immunostaining area (%) of Masson’s Trichrome. Data shown as mean ± s.e.m., error bars show the variations of determinations in terms of standard error. One-way analysis of variance, followed by Tukey’s post hoc analysis using GraphPad Prism software (USA, version 8.0.1.) was used for data analysis (n = 5), values with unlike superscript letters were significantly different (*p* < sss0.05).

Masson’s Trichrome staining was performed to assess fibrosis, (Figs. 10 and 11). Histopathological examination showed extensive perivascular fibrosis in diabetic group, in contrast to normal group. Treatment with different extracts showed marked improvement in fibrosis with best result observed in EE group, which had the same picture as that of the normal, followed by that of BE with minimal fibrosis. TE and GZ-treated groups exhibited moderate fibrosis.

The outcomes of the current investigation unveiled that the highest level of phenolic content in dry ripe fruit of OFI-RF was detected in the butanol fraction, followed by the ethyl acetate fractionand finally in the total alcohol fraction (148.91 ± 0.95, 110.96 ± 0.61, and 93.07 ± 2.57 mg GAE (gallic acid equivalents)/g of fruit pulp for total phenolics, respectively). Similarly, the highest concentration of flavonoids was found in the butanol fraction, followed by ethyl acetate and methanol extracts (31.10 ± 1.07, 20.41 ± 0.16, and 11.47 ± 0.74 mg QE (quercetin) equivalents/g OFI-RF for total flavonoids, respectively). These results align with the research by Mohammed et al.^52^. Antidiabetic and antioxidant activities conducted in vitro and in vivo could be attributed to rich fractions in total phenol and flavonoid content (Table 1S and Fig. 1).

Metabolomics, facilitated by high-throughput and sensitive UPLC-HESI-MS/MS, has facilitated comprehensive investigations into various secondary metabolites present in different fractions of OFI-RF. This analysis unveiled a total of 101 distinct compounds, predominantly comprising phenolics, flavonoids, betalains, alkaloids, and nine unique fatty acids. Identification of individual compounds involved precise determination of molecular masses with a margin of error less than 5 ppm, along with analysis of mass spectra and retention times in comparison to standard compounds. Additionally, various databases including ChEBI, Metlin, PubChem, and KNApSAck, as well as literature data, were utilized for compound identification. Enhancements in annotation coverage were achieved through the development of reference MS and MS/MS databases, complemented by a query data search algorithm. This algorithm facilitated comparison of m/z values in query data with reference data obtained from authentic compounds, resulting in the retrieval of matched data within user-defined tolerance limits for both unit and high-resolution m/z values. The MS/MS search algorithm leveraged both m/z values and intensities for scoring the probability of a match between query and reference data. Furthermore, untargeted data analysis allowed for tolerance adjustments in both chromatographic retention time and m/z values, with drifts across measurements being rectified through the utilization of alignment software MS-DIAL. To streamline the process from data processing to analysis, automated workflows encompassing tasks such as peak alignment of raw data, annotations, statistical analysis, and visualization have been implemented^53^. These workflows are accessible through advanced web-based platforms, enabling efficient annotation of numerous MS data sets and catering to a diverse audience of researchers.

**Fig. 12:**
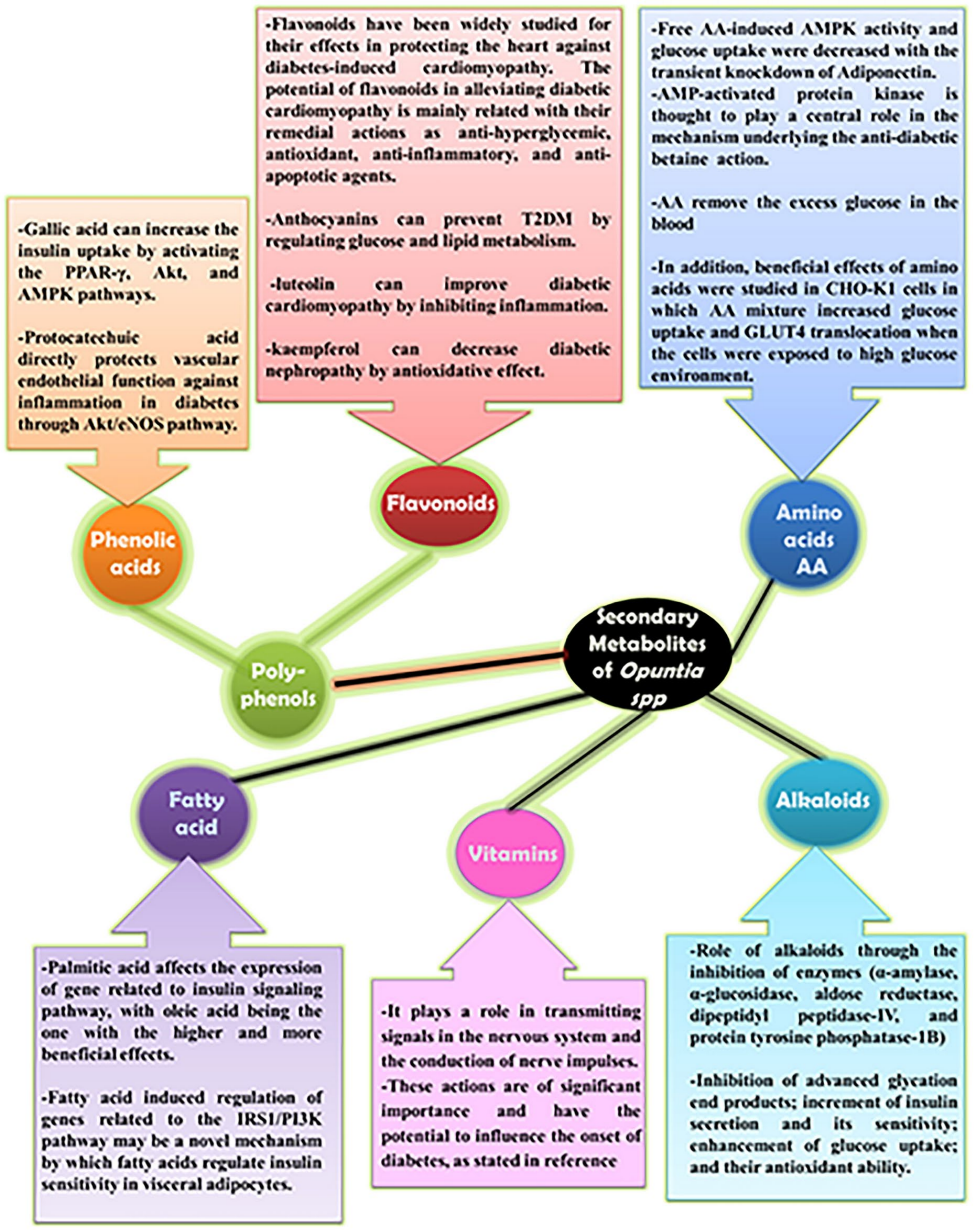
Role of secondary metabolites from *Opuntia* spp with effects against diabetes and related complications^54–60^.

In the current work, a comprehensive characterization of the secondary metabolites using LC–MS/MS was accomplished in the hydroalcoholic extract along with its methanol, ethyl acetate, butanol fractions as well as the total alcohol. The analysis revealed 101 secondary metabolites, divided into eight groups: *Group 1*: phenolic acids and phenolic glycosides; *Group 2*: flavanols, flavanonols, and flavonoids; *Group 3*: betalains, alkaloids, and pyridine carboxylic acids; *Group 4*: amino acids and derivatives; *Group 5*: carboxylic acids, *Group 6*: coumarins; *Group 7*: organic compounds*;* and* Group 8:* fatty acid profiles. Total flavonoid and phenolic contents were more pronounced in the BE and EE extracts, hence, both extracts exhibited potential antioxidant, antidiabetic and antihyperlipidemic activities. Different types of phenolic compounds, including phenolic acids and polyphenols, such as quinic acid, gallic acid, cinnamic acid, piscidic acid derivative II, piscidic acid, coumaric acid, vanillic acid, protocatechuic acid, *p*-hydroxybenzoic acid, benzoic acid, benzoic acid glucoside, caffeoylquinic acid, trans-caffeic acid, syringic acid, 4-methoxycinnamic acid, 3-o-feruloylquinic acid, p-coumaric acid, ferulic acid, sinapic acid, vanillin and methoxyphenols (syringaldehyde and feruloyltyramine) and rhoifolin, isoquercetrin, isorhamnetin 3-galactoside, diosmin, cyanidin-3-o-galactoside, kaempferol-3-o-rutinoside, acacetin-7-o-neohesperidoside, naringin, hyperoside, tiliroside, rutin, kaempferol 7-o-glucoside, guajavarin, isorhamnetin-3-o-rutinoside, narcissin, isorhoifolin, cynaroside, isorhamnetin 3-galactoside, dihydrokaempferol, flavanonols, cynaroside, quercetin, naringenin, formononetin, baicalein, jaceidin, cirsiliol, kaempferol, kaempferol 3-o-methyl ether, isokaempferide, taxifolin, cirsimaritin, isorhamnetin, 5-hydroxy-6,7-dimethoxyflavone, skullcapflavone II, neobaicalein, acacetin and other metabolites were identified from the OFI-RF phenolic extract by different organic solvents (Table 1). Figures 4 and 12 illustrates a concise pathway delineating the protective effects of all identified compounds in OFI-RF dry ripe fruit against hyperglycemia, reactive oxygen species (ROS) and hyperlipidemia, along with the proposed mechanisms documented in existing literature^54–60^ in Figures S1-S5.

**Fig. 13:**
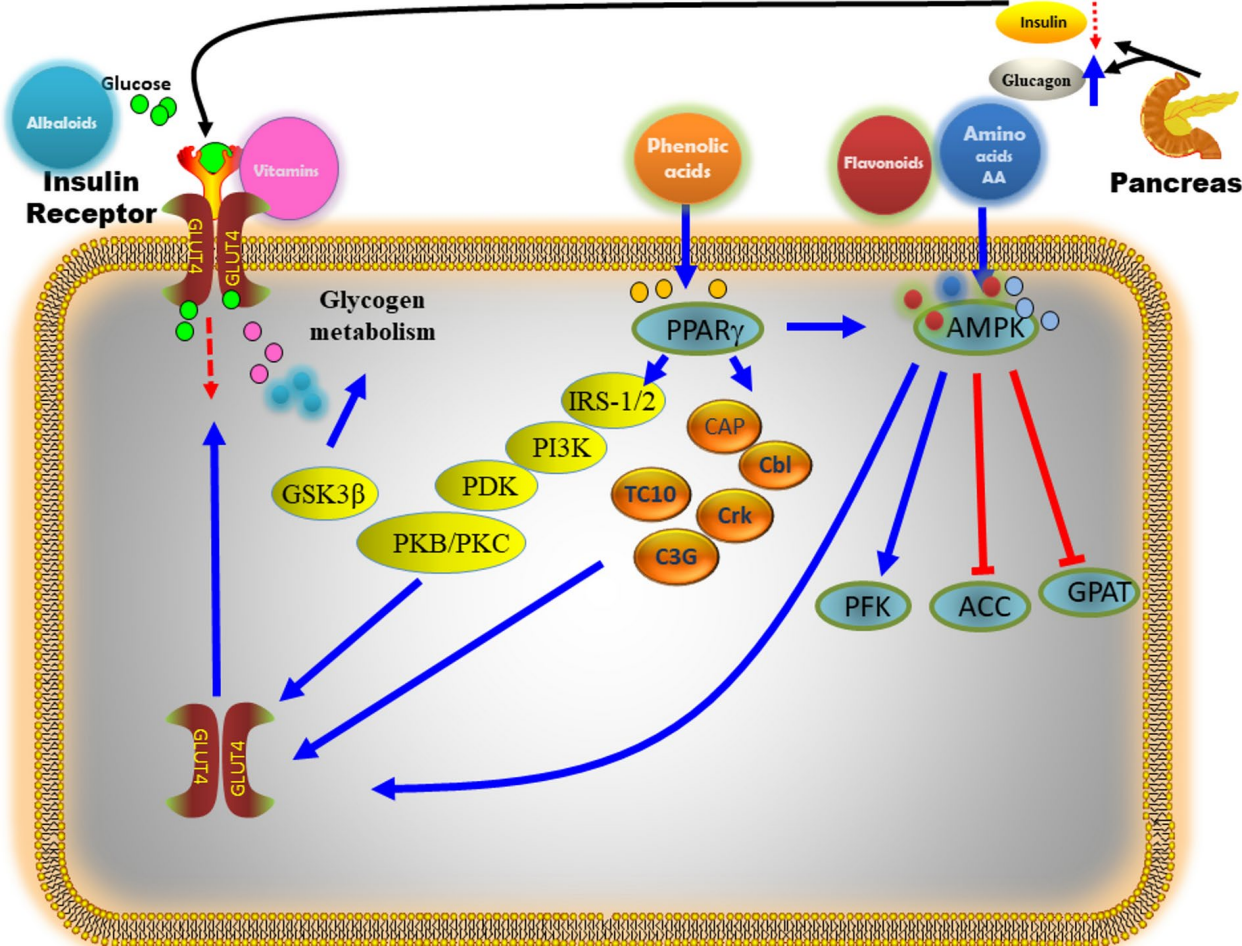
Pharmacological pathways of OFI-RF secondary metabolites involved in glucose homeostasis.

Phenolic acids are characterized as aromatic carboxylic acids containing hydroxyl derivatives, featuring a singular phenolic ring within their chemical structure. These acids encompass two categories: hydroxybenzoic and hydroxycinnamic acid derivatives^61^. Hydroxycinnamic acid derivatives such as; caffeic, *p*-coumaric, ferulic and sinapic acids are more prevalent in plant sources as compared to benzoic acid derivatives (gallic, protocatechuic and *p*-hydroxybenzoic acids). Biological activities are depicted with proposed mechanistic action in Table 1 and Figs. 4, 12 and 13).

The pathway illustrated in this diagram highlights key molecular signaling processes involved in glucose metabolism, insulin signaling, and energy regulation. When insulin binds to its receptor, it initiates a cascade that promotes glucose uptake into cells, particularly in muscle and fat tissue. This is facilitated by GLUT4 (Glucose Transporter Type 4), which moves to the cell membrane to allow glucose entry. Insulin signaling also triggers various proteins such as IRS-1/2 (Insulin Receptor Substrate-1/2) and PI3K (Phosphoinositide 3-Kinase), which further propagate the signal^62^.

The pathway includes PKB/PKC (Protein Kinase B/Protein Kinase C) and PDK (Phosphoinositide-dependent Kinase), which enhance glucose uptake and promote glycogen synthesis by inhibiting GSK3β (Glycogen Synthase Kinase 3 Beta), an enzyme that would otherwise hinder glycogen production. Additionally, proteins like TC10, CAP (Cbl-associated Protein), Cbl, Crk, and C3G are part of this signaling cascade, helping to regulate glucose transport by modulating cytoskeletal organization.

Bioactive compounds such as phenolic acids, flavonoids, alkaloids, amino acids, and vitamins interact with this pathway, influencing molecules like PPARγ (Peroxisome Proliferator-Activated Receptor Gamma) and AMPK (AMP-Activated Protein Kinase). Activation of PPARγ, often induced by phenolic acids, plays a role in lipid and glucose metabolism, improving insulin sensitivity. Similarly, AMPK activation by amino acids and other natural compounds regulates energy balance by controlling enzymes such as PFK (Phosphofructokinase), **ACC** (Acetyl-CoA Carboxylase), and GPAT (Glycerol-3-Phosphate Acyltransferase), reducing lipid synthesis and promoting fat oxidation^63^. In essence, this pathway underscores how insulin and various signaling proteins, in conjunction with bioactive compounds, contribute to glucose uptake, glycogen metabolism, and overall metabolic health.

Polyphenolic compounds, as their name implies, are distinctive due to the presence of various phenolic groups, which can be connected to low or high molecular weight groups of chemicals to construct their molecular configurations. These compounds are products of plant metabolism^64^. The quantity and arrangement of hydroxyl groups in a specific phenolic compound result in diverse variations in their antioxidative capacity^65^, so they could represent an essential source of dietary antioxidants that are readily assimilated within the gastrointestinal tract^66^. Therefore, polyphenols are widespread and have been extensively recognized for their additional health-protective benefits (Figs. 6 and 7). The mechanism proposed for radical scavenging could be explained by the phenolic group and resonance-stabilized structure in phenolic acids, which facilitate the donation of hydrogen atoms. An additional mechanism of antioxidant capacity involves the suppression of radicals through donation of electrons and the quenching of singlet oxygen^67^. This information was consistent with the antioxidant potential demonstrated in vitro and in vivo by the selected extracts, with superior effect observed from the EE and BE of *Opuntia* spp. fruit (Fig. 5).

Phenolic acids also exhibited a characteristic feature against diabetes and its complications as recorded in the current in vivo studies possibly via activation of the PPAR-γ, Akt and AMPK pathways by gallic acid, which may enhance insulin uptake^68^ in Fig. 13. The regulation of TNF and adipocytokines expressions may also be managed to amplify the antidiabetic effects of gallic acid, which could eventually protect against β-cell death and improve the functionality of the pancreatic islets^69,70^. Chlorogenic and ferulic acids, found in selected extracts of this study, showed similar transporter stimulation mechanisms and exhibited antidiabetic properties^71^ in Figs. 4 and 13. Various mechanisms proposed for phenolic acids in diabetes have been outlined in literature reviews^59,72^.

Betaine therapy enhanced glucose tolerance and insulin action, particularly impacting insulin-sensitive tissues such as skeletal muscle, adipose tissue and liver^55^. The positive impact of betaine supplementation extends to multiple genes, the dysregulation of which is associated with diabetes. The antidiabetic action of betaine was believed to be mediated by AMP-activated protein kinase^55^. Furthermore, research involving animal models of type 2 diabetes has indicated that betaine exhibits anti-inflammatory/antioxidant properties and mitigates endoplasmic reticulum stress^73^. These alterations contribute to heightened insulin sensitivity and improved blood glucose metabolism. Findings from animal studies may pave the way to further investigate therapeutic efficacy of betaine in individuals with type 2 diabetes^55^. Quercetin attached sugar is removed before absorption, either by lactase phloridzin hydrolase in the small intestine or by the gut microbiota^74^.

## Conclusion

Metabolomic analysis using UPLC-HESI-MS/MS identified 101 distinct compounds that consisted of phenolics, flavonoids, betalains and fatty acids among other different OFI-RF fractions. Findings of this study suggested that high content of polyphenolics and flavonoids in BE and EE could serve as natural antioxidants with potential applications in food and nutraceuticals. Additionally, the study highlighted the diverse mechanisms through which phenolic acids and other compounds in the selected extracts of *Opuntia spp.* exhibited remarkable effects against diabetes and its related complications, such asinhibition of key enzymes involved in glucose homeostasis *(*in vitro*)*. Moreover, both extracts revealed marked improvement in carbohydrate metabolism with superior effect observed in BE-treated groups. Similar findings were noticeable against diabetes-induced oxidative stress and hyperlipidemia Future research is recommended in order to study the long-term daily consumption of *Opuntia* spp red fruits (OFI-RF) and confirm being a potential cost-effective nutritional intervention for the early protection against T2DM with associated complications, since quantifiable amounts of beneficial polyphenolics was obtained optimally in butanol and ethyl acetate extracts.

## Supplementary Information

Supplementary Information.

## Data Availability

The datasets used in this investigation are all included in this publication, and other information files are added.

## Abbreviations

ABTS: 2,2-Azino-bis-3-ethylbenzothiazoline-6-sulfonic acid
BE: Butanol extract
BuOH: Butanol
CE: Chloroform extract
DPPH: 2,2-Diphenylpicrylhydrazyl
EE: Ethylacetate extract
ESI: Electrospray ionization
EtOAc: Ethyl acetate
GZ: Gliclazide
HDL: High density lipoprotein
HPLC: High performance liquid chromatography
LC/MS/MS: Liquid chromatography–electrospray ionization–tandem mass spectrometry
LDL: Low density lipoprotein
MDA: Malondialdahyde
OFI-RF: *Opuntia* Spp. red fruits
PE: Petroleum ether extract
QC: Quality control
SOD: Superoxide dismutase
STZ: Streptozotocin
T2DM: Type 2 diabetes mellitus
TAC: Total antioxidant capacity.
TE: Total extract
UPLC/HESI-MS/MS: Ultra-performance liquid chromatography-electrospray ionization-tandem mass spectrometry
