# Supplementary material for: Role of rich phenolics and betanin profiles from Opuntia ficus-indica fruits in the prevention of diabetic complications using metabolomics study
Source: Sci Rep. 2025 Feb 17;15:5780. doi: 10.1038/s41598-024-81874-1 (PMC11833125; doi:10.1038/s41598-024-81874-1)
Supplement: Supplementary file 1 — Supplementary Information. [file 41598_2024_81874_MOESM1_ESM.docx]

**Article type**: Original Article

**Title: Role of rich phenolics and betanin profiles from *Opuntia ficus-indica* fruits in the prevention of diabetic complications using metabolomics study**

Mona A. Mohammed^1*#^, Souad E. El-Gengaihi^1^, Yousreya A. Maklad^2^, Marwa E. Shabana^3^, Hanan Naeim Attia^2#^

^1^Medicinal and Aromatic Plants Research Department, Pharmaceutical Industries Research Institute, National Research Centre, Giza, Egypt.

^2^Medicinal and Pharmaceutical Chemistry Department (Pharmacology group), Pharmaceutical Industries Research Institute, National Research Centre, Giza, Egypt.

^3^Pathology Department, Medical Research and Clinical Studies Institute, National Research Centre, Giza, Egypt.

**# These authors contributed equally to this work.**

***Corresponding Authors**

Mona A. Mohammed ([monaarafamohammed@yahoo.com](mailto:monaarafamohammed@yahoo.com), [on.ibrahim@nrc.sci.eg](mailto:on.ibrahim@nrc.sci.eg))

**Table S1**

Total phenolic and flavonoid content, *in vitro* anti-diabetic and antioxidant activities of Opuntia extracts and fractions

| **Extracts** | **Rich total Phenolics** | | **Anti-diebatics Enzymes** | |
| --- | --- | --- | --- | --- |
|  | **Phenolic content** | **Flavonoid content** | **α-amylase** | **α-glucosidase** |
|  | **mg gallic acid /g extract** | **mg rutin/g extract** | **(IC_50_, µg/mL)** | |
| **Pet. ether** | 0 | 0 | 50.28 ± 0.03 | 55.33 ± 1.00 |
| **CHCL3** | 20.67 ± 0.56 | 1.89 ± 0.05 | 45.67 ± 0.57 | 50.73 ± 0.55 |
| **EtOAc** | **110.96 ± 0.61** | **20.41 ± 0.16** | **7.51 ± 0.17** | **8.33 ± 0.02** |
| **BuOH** | **148.91 ± 0.95** | **31.10 ± 1.07** | **6.51 ± 0.24** | **5.58 ± 0.45** |
| **H2O** | 88.67 ± 2.87 | 3.38 ± 0.23 | 31.06 ± 1.33 | 29.76 ± 0.51 |
| **Total alcohol** | **93.07 ± 2.57** | **11.47 ± 0.74** | **10.29 ± 0.33** | **10.99 ± 0.58** |
| **Acarbose (SD)** | - | - | 2.5 ± 0.3 | 4.5 ± 2.7 |

**Table S2.**

In vitro DPPH antioxidant activity of different extracts

| **DPPH/ Conc Ug/ml** | **Pet. ether** | **CHCL_3_** | **EtOAc** | **BuOH** | **H2O** | **Total alcohol** |
| --- | --- | --- | --- | --- | --- | --- |
| **20** | 80.48 ± 0.10 | 80.71 ± 0.0 | 95.25 ± 0.05 | 96.46 ± 0.3 | 79.37 ± 0.07 | 89.74 ± 0.23 |
| **15** | 75.62 ± 0.07 | 73.16 ± 0.0 | 85.27 ± 0.04 | 90.2 ± 0.07 | 64.4 ± 0.12 | 83.85 ± 0.07 |
| **10** | 43.57 ± 0.07 | 62.06 ± 4.17 | 77.45 ± 0.07 | 80.16 ± 0.04 | 56.47 ± 0.07 | 78.93 ± 0.07 |
| **5** | 30.28 ± 0.05 | 52.86 ± 2.01 | 55.73 ± 0.06 | 75.94 ± 0.04 | 30.25 ± 0 | 77.7 ± 0 |
| **2.5** |  | 40.25 ± 0.01 | 40.53 ± 0.01 | 70.21 ± 0.06 |  | 60.27 ± 0.04 |
| **1** |  |  | 27.69 ± 0.19 | 60.3 ± 0.04 |  | 42.34 ± 0.02 |
| **0.5** |  |  | 10.28 ± 0.05 | 40.42 ± 0.07 |  |  |
| **IC_50_** | 48.23 | 18.74 | **5.786** | **1.447** | 10.2 | **1.352** |

**Table S3.**

In vitro ABTS+ antioxidant activity of different extracts *Opuntia* Extracts.

| **ABTS/ Conc Ug/ml** | **Pet. ether** | **CHCL3** | **EtOAc** | **BuOH** | **H2O** | **Total alcohol** |
| --- | --- | --- | --- | --- | --- | --- |
| **20** | 81.52 ± 0.25 | 82.54 ± 0.25 | 97.26 ± 0.07 | 98.46 ± 0.3 | 79.37 ± 0.07 | 89.74 ± 0.23 |
| **15** | 76.62 ± 0.18 | 75.49± 0.28 | 89.21 ± 0.15 | 92.16 ± 0.07 | 74.4 ± 0.12 | 85.72 ± 0.17 |
| **10** | 41.57 ± 0.09 | 65.93± 5.81 | 78.54 ± 0.14 | 82.16 ± 0.04 | 56.47 ± 0.07 | 79.59 ± 0.4 |
| **5** | 32.65 ± 0.01 | 54.86 ± 0.47 | 59.45 ± 0.32 | 79.93 ± 0.04 | 32.65 ± 0 | 78.29 ± 0.52 |
| **2.5** |  | 41.25± 0.02 | 50.27 ± 0.04 | 65.3 ± 0.04 |  | 62.37 ± 0.06 |
| **1** |  |  | 29.67 ± 0.14 | 45.87 ± 0.15 |  | 45.4 ± 0.18 |
| **0.5** |  |  | 12.28 ± 0.05 |  |  |  |
| **IC_50_** | 120.5 | 10.89 | 4.02 | 2.243 | 6.933 | 1.434 |

**Metabolomics and molecular networking data**

( Q-Exactive MS operated upon following settings: the HESI ion source voltage -3kV or 3kV. The sheath gas (N2) flow 48 l/min, auxiliary gas flow 13 l/min, ion source capillary temperature 250 °C, auxiliary gas heater temperature 380 °C. The CID MS/MS experiments were performed using collision energy of 15 eV. [2, 3]).

Fig S1.

LCMSMS chromatogram,**tentative mass in successive extract fractionation (methanol (black); butanol (red); ethyl acetate(green) and QC sample (blue))**


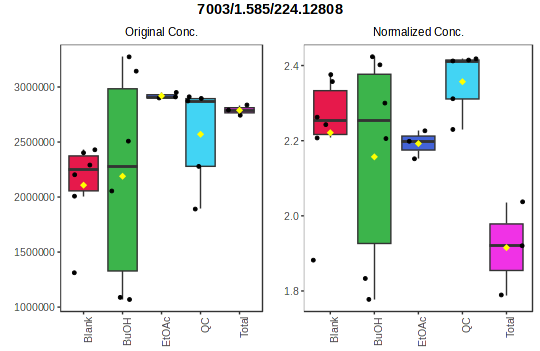


Fig S2. Compound of 4-Hydroxybenzoylcholine with differ concentration in different fraction.


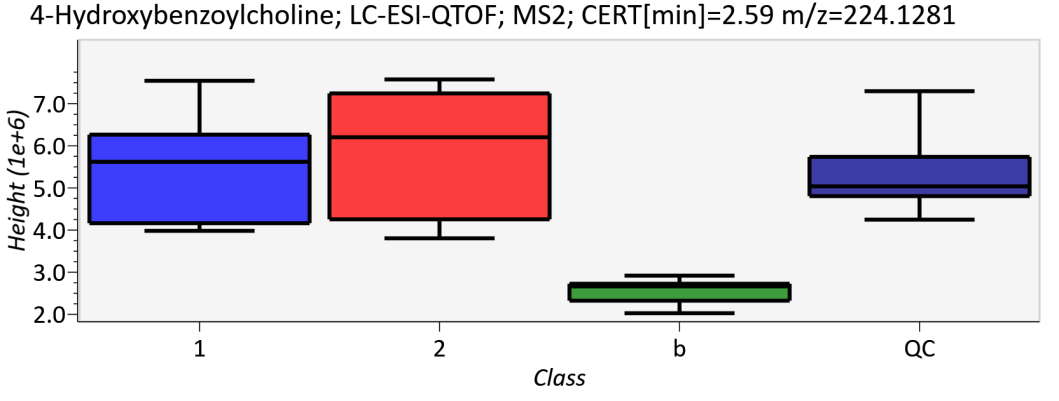

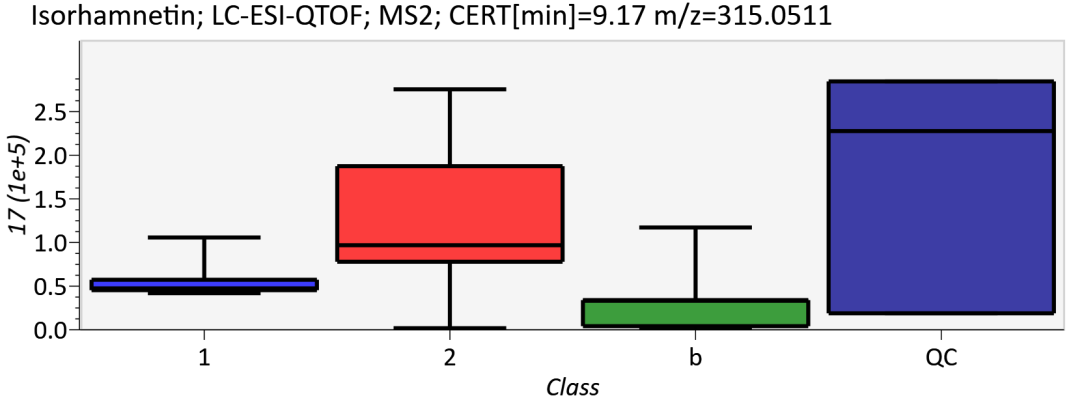

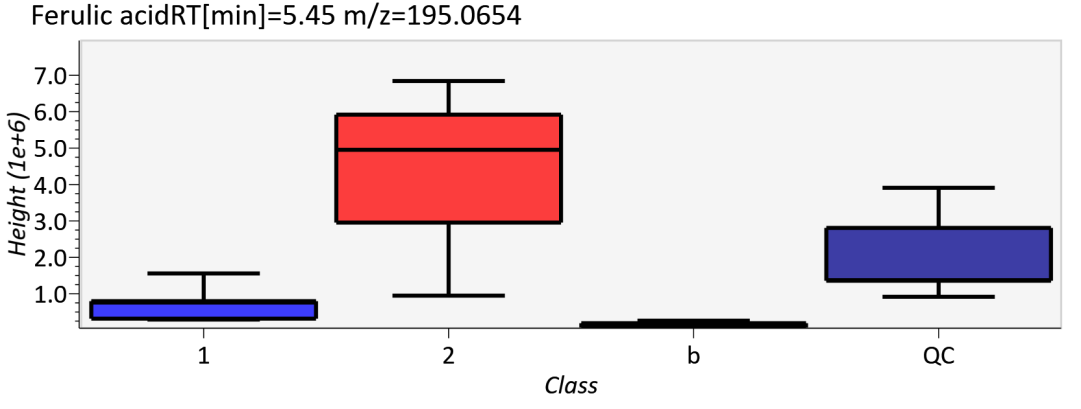


**Fig S3 different conc of compounds**


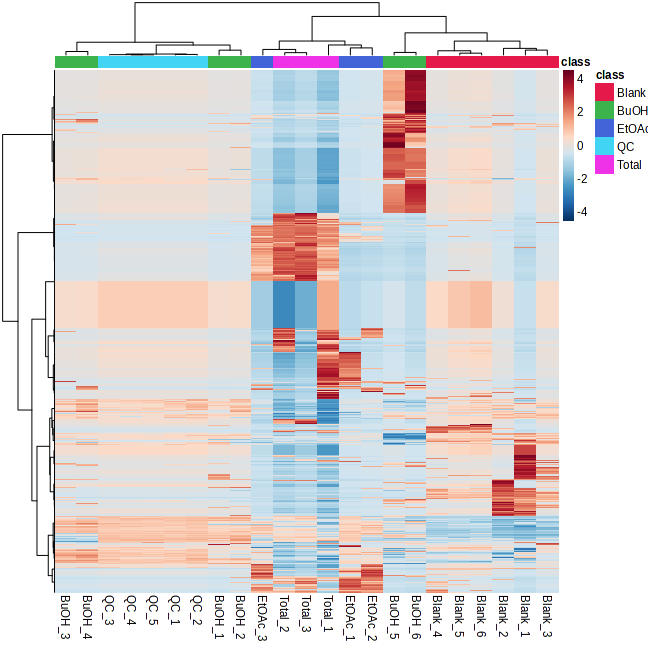


Fig S4 Heatmap showed in negative mode between different extract of *Opuntia* spp recorded in clusters from grouping of fractionated related to different class of compounds.


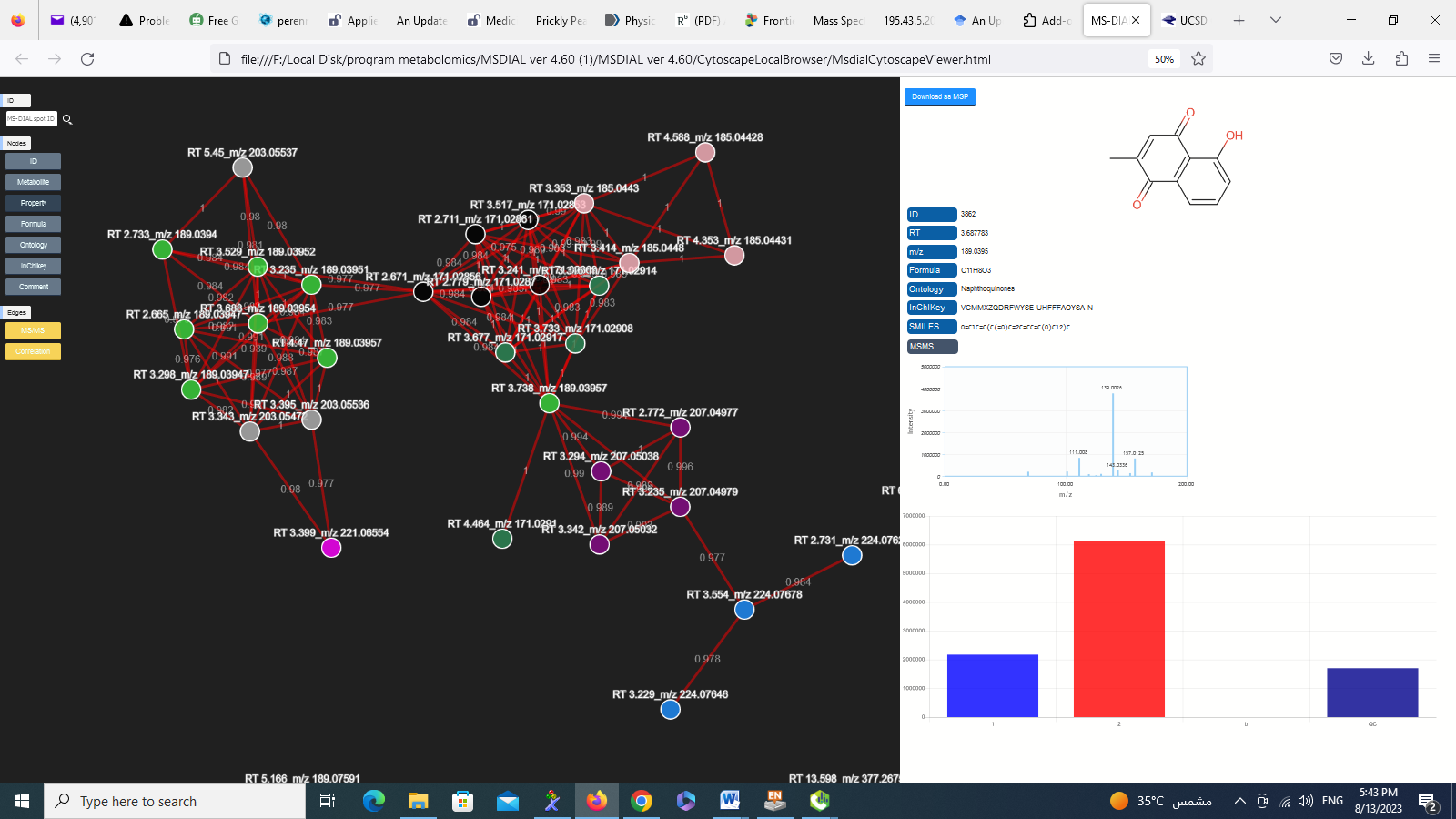

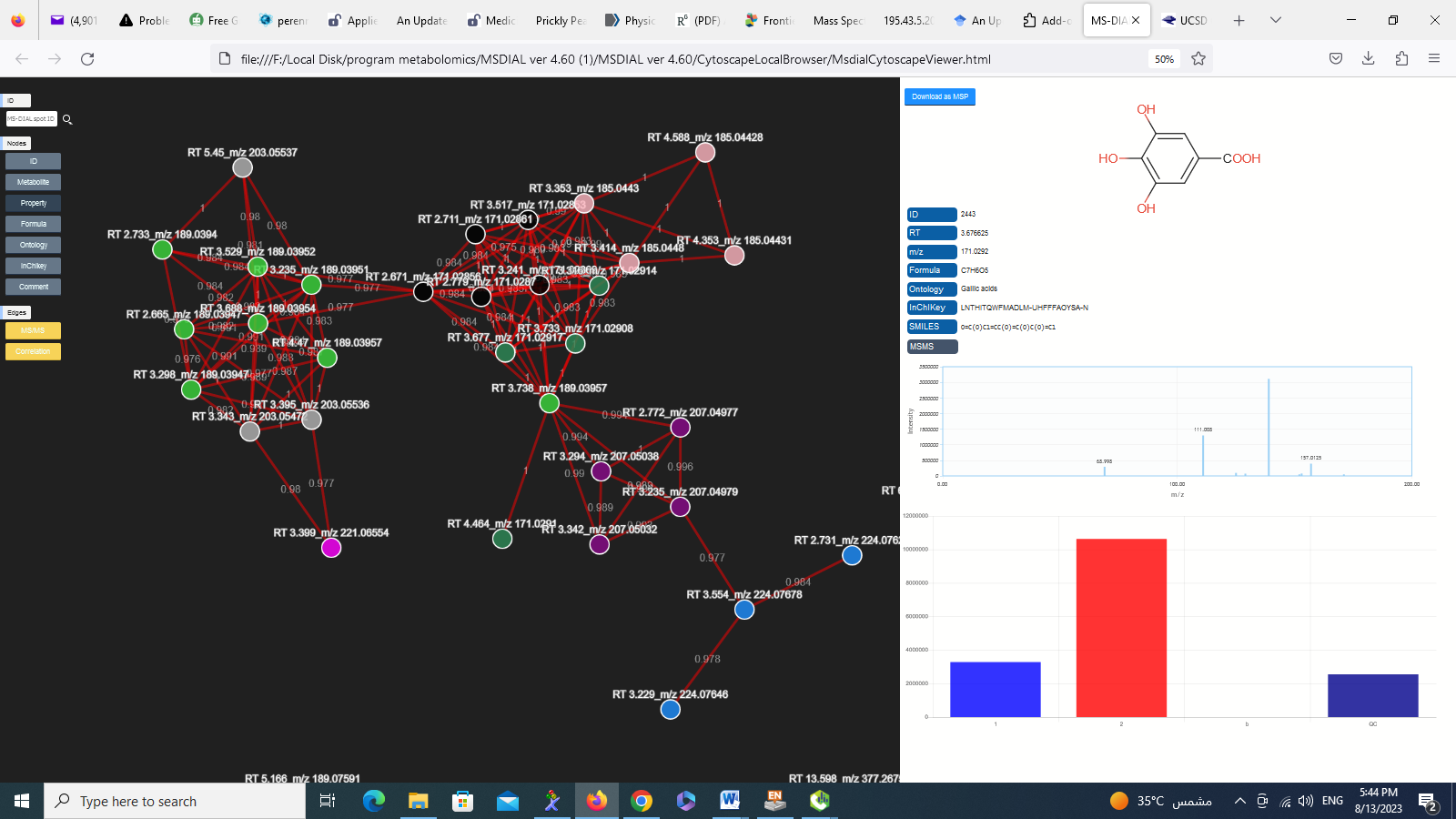


Fig S5 Methods for the generation of molecular networks from metabolomics mass spectrometry using MS-DIAL 4.36 with URL link ([MS-DIAL molecular spectrum networking](file:///F:\Local%20Disk\program%20metabolomics\MSDIAL%20ver%204.60%20(1)\MSDIAL%20ver%204.60\CytoscapeLocalBrowser\MsdialCytoscapeViewer.html)) from data visualization, then molecular spectrum networking data with the GNPS web platform (<http://gnps.ucsd.edu/ProteoSAFe/status.jsp?task=50e1da857cae47ac8fc696a9852a124b>) for all signals and showed gallic acid and coumarin compound.

**Materials and Methods**

**Antioxidants activity of plant extracts.**

**a. DPPH free radical-scavenging activity**

The DPPH (1,1-diphenyl-2-picrylhydrazyl, 250 mM) radical scavenging model of Shimada *et al*. (1992) was used. 2 ml of 100 µM DPPH- solution in ethanol was mixed with 2 ml of 100µg/ml each extract. The effective test concentrations of DPPH- and the extracts were 50µM and 50µg/ml, respectively. The reaction mixture with different dilutions, for different concentrations, was incubated in the dark for 30 minutes and there after the optical density was recorded at 517 nm against blank. For control, 2 ml of methanol was added instead of plant mixture and run simultaneously with the test. Inhibition (%) was plotted against the extract concentration in the reaction system. The percentage inhibition of the DPPH radical was calculated according to the following formula:

% Inhibition = [(A control −A sample) / A control] X 100

Where A is absorbance at 517nm.

**b. ABTS^+^ Radical Cation Scavenging Activity**

ABTS was dissolved in water to a 7 mM concentration. ABTS radical cation (ABTS**^•+^**) was produced by reacting ABTS stock solution with 2.45 mM potassium persulfate (final concentration) and allowing the mixture to stand in the dark at room temperature for 12–16 h before use because ABTS and potassium persulfate react stoichiometrically at a ratio of 1:0.5, this will result in incomplete oxidation of the ABTS. Oxidation of the ABTS commenced immediately, but the absorbance was not maximal and stable until more than 6 h had elapsed. The radical was stable in this form for more than two days when stored in the dark at room temperature. For the study of phenolic compounds and food extracts, the ABTS**^•+^** solution was diluted with ethanol for plant extract plasma antioxidants with PBS, pH 7.4, to an absorbance of 0.70 (± 0.02) at 734 nm and equilibrated at 30°C. Stock solutions of phenolics, flavonoids, alkaloids and etc. in water (H_2_O), butanol (BE), ethylacetate (EE), chloroform (CE), petroleum ether (PE), and total extract (TE) were diluted such that, after introduction of a 10 ml aliquot of each dilution into the assay, they produced between 20%–80% inhibition of the blank absorbance. After addition of 1.0 ml of diluted ABTS^•+^ solution (A_734nm_ = 0.700 ± 0.020) to 50 µl of antioxidant compounds or Trolox standards (final concentration 0–15mM) in ethanol or PBS the absorbance reading was taken at 30°C exactly 1 min after initial mixing and up to 6 min. Appropriate solvent blanks were run in each assay Dinkova-Kostova et al [27]

All determinations were carried out at least three times, and in triplicate, on each occasion and at each separate concentration of the standard and samples. The percentage inhibition of absorbance at 734 nm is calculated and plotted as a function of concentration of antioxidants and of Trolox for the standard reference data. The concentration response curve for 5 sequentially and separately prepared stock standards of Trolox and Vit C.
